# Relationship between Vitamin B12 and Cobalt Metabolism in Domestic Ruminant: An Update

**DOI:** 10.3390/ani10101855

**Published:** 2020-10-12

**Authors:** Jose-Ramiro González-Montaña, Francisco Escalera-Valente, Angel J. Alonso, Juan M. Lomillos, Roberto Robles, Marta E. Alonso

**Affiliations:** 1 Medicine, Surgery and Anatomy Veterinary Department, Veterinary Faculty, University of León, 24071 León, Spain; ajalod@unileon.es (A.J.A.); roblesdellano@gmail.com (R.R.); 2 Academic Unit of Veterinary Medicine and Zootechnics, Autonomous University of Nayarit, Tepic 69130, Nayarit, Mexico; fescalera@uan.edu.mx; 3 Production and Health Animal, Public Health Veterinary and Science and Technology of Food Department, Veterinary Faculty, Cardenal Herrera-CEU University, 46115 Valencia, Spain; juan.lomillos@uchceu.es; 4 Animal Production Department, Veterinary Faculty, Veterinary Faculty, University of León, 24071 León, Spain; marta.alonso@unileon.es

**Keywords:** cobalt, vitamin B12, deficiency, ruminant, cattle

## Abstract

**Simple Summary:**

We review the role of cobalt and vitamin B12 in animals, especially in ruminants. Vitamin B12 is an essential part of the enzyme systems involved in multiple metabolic reactions and mainly in the formation of energy from ruminal fermentation. Signs of deficiency, as well as cobalt toxicity, in animals are described. The level of cobalt in ruminants can be assessed by measuring the blood or tissue concentrations of cobalt or vitamin B12, as well as the level of some metabolites such as malonate, homocysteine or transobolamine in blood or methylmalonic acid in urine. The requirement for cobalt (Co) is around 0.11 ppm (mg/kg) dry matter (DM) in the diet, although current recommendations advise supplementing the diet up to 0.20 mg Co/kg DM, which seems to increase animal production, especially in dairy cattle.

**Abstract:**

Cobalt, as a trace element, is essential for rumen microorganisms for the formation of vitamin B12. In the metabolism of mammals, vitamin B12 is an essential part of two enzymatic systems involved in multiple metabolic reactions, such as in the metabolism of carbohydrates, lipids, some amino acids and DNA. Adenosylcobalamin and methylcobalamin are coenzymes of methylmalonyl coenzyme A (CoA) mutase and methionine synthetase and are essential for obtaining energy through ruminal metabolism. Signs of cobalt deficiency range from hyporexia, reduced growth and weight loss to liver steatosis, anemia, impaired immune function, impaired reproductive function and even death. Cobalt status in ruminant animals can be assessed by direct measurement of blood or tissue concentrations of cobalt or vitamin B12, as well as the level of methylmalonic acid, homocysteine or transcobalamin in blood; methylmalonic acid in urine; some variables hematological; food consumption or growth of animals. In general, it is assumed that the requirement for cobalt (Co) is expressed around 0.11 ppm (mg/kg) in the dry matter (DM) diet; current recommendations seem to advise increasing Co supplementation and placing it around 0.20 mg Co/kg DM. Although there is no unanimous criterion about milk production, fattening or reproductive rates in response to increased supplementation with Co, in some investigations, when the total Co of the diet was approximately 1 to 1.3 ppm (mg/kg), maximum responses were observed in the milk production.

## 1. Introduction

Cobalt (Co) is a metallic element with an atomic weight of 58.9. It is considered an essential trace element, because it is required in the human diet and of some animal species in very small amounts, close to 100 mg per kg of dry matter [1,2,3]. As such, cobalt has no known nutritional function, except as a component of vitamin B12, so when we refer to the Co status, we are really referring to the vitamin B12 status [4,5].

It is known that cobalt (Co), in ruminants, is an essential component for the microbial synthesis of vitamin B12, a water-soluble vitamin belonging to group B, commonly known as cobalamin, cyanocobalamin or also called the pernicious antianemia factor [6]. Although, technically, vitamin B12 refers only to cyanocobalamin, actually, the term vitamin B12 is the generic name used to refer to a group of compounds that have B12 activity, such as cyano, hydroxy, methyl or deoxyadenosylcobalamin, and which are also known as complete corrinoids [7,8]. There are many different analogs and derivatives devoid of biological activities [8,9], and there are even different isoforms of cobalamin (CBL) [7].

**Figure 1:**
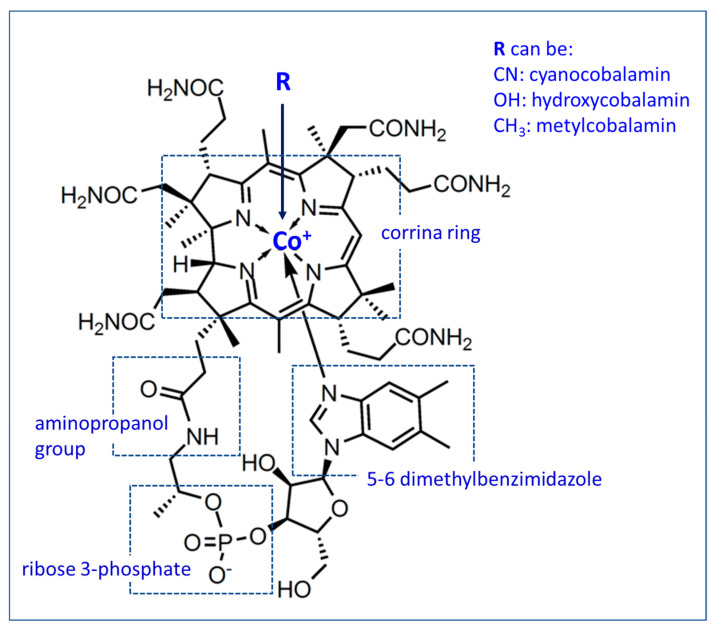
Empirical formula of vitamin B12.

All of them present very complex structures, being the chemically largest of all vitamins, with a molecular weight of 1355 and whose empirical formula is C_63_H_88_O_14_N_14_PCo. One of its main characteristics is to present a cobalt content between 4.4% and 5.8% [1,8,10,11,12]. The molecule is made up of four main parts: the corrin ring (similar to that found in heme, chlorophyll and cytochromes); the remaining nucleotides; the aminopropanol residue that binds the nucleotide to the corrin ring and the ligand B attached to the cobalt atom as the central nucleus of the corrin ring [12,13,14,15,16]. In cyanocobalamin (CBL), 5,6-dimethylbenzimidazole (5,6-DMB) is the base in the nucleotide fraction (Figure 1) [17].

Although cobalt is known as an essential trace element for humans and animals, there are differences in the way it must be supplied in the diet of different species. While vegetables cannot synthesize this vitamin, humans and most monogastric animals (exclusive animals with cecotrophy or coprophagia) require cobalt in its active form (vitamin B12). However, in adult ruminants, vitamin B12 is produced during the microbial fermentation of food in the stomachs and, mainly, in the rumen [18]. The ruminal flora—that is, the microorganisms, bacteria and yeasts present in the rumen—can synthesize vitamin B12, provided that the cobalt concentration in the ruminal fluid is higher than 0.5 mg/mL, while if this level is not reached, the ruminal synthesis of vitamin B12 remains inhibited, reducing its contribution to blood and other tissue [6,9,19,20].

Even the ciliated protozoa present in the rumen need vitamin B12, which they obtain from ruminal bacteria that synthesize vitamin B12 [17]. In addition, these bacteria, present in the rumen, use dietary cobalt to produce vitamin B12 analogs, molecules chemically related to cyanocobalamin but devoid of biological activity [17,21]. The production of vitamin B12 by ruminal microflora, as with folates, is generally considered sufficient to avoid deficiency symptoms in ruminants [12,22], although, in steers, it has been shown that the ruminal microflora extensively destroys dietary folic acid and vitamin B12 [22,23].

As early as 1935, cobalt was shown to be an essential nutrient for ruminants when it was discovered that it corrects a disorder characterized by reduced appetite and weight loss [6]. A few years later, in 1948, it was found that cobalt was an essential component of vitamin B12 for sheep and cattle, and its lack caused conditions such as coastal disease (in sheep), wasting disease or enzootic marasmus in cattle [6]. Vitamin B12 deficiency is associated with conditions such as methylmalonic aciduria, megaloblastic anemia and pernicious anemia [6]. Its deficiency has been described in various regions around the world (Australia: Marston in 1935, New Zealand: McNaught in 1948, United States: Ammermann in 1969 and tropical regions: McDowell et al. in 1993). Animals with vitamin B12 deficiency show nonspecific clinical symptoms, such as reduced food intake, retarded growth, muscle wasting, rough coat and thickening of the skin. Reproductive disorders and decreased milk yield are often observed [4,6].

In young ruminants (preruminant lambs and calves) up to the ages of six to eight weeks, the rumen is not fully developed and not functional for the synthesis of this vitamin [18]. Therefore, they require a dietary source of vitamin B12, such as colostrum, milk or milk replacers [18,20]. In contrast, adult domestic ruminants do not necessarily depend on a dietary source of vitamin B12, because ruminal microorganisms are capable of synthesizing vitamin B12 from Co [24]. The efficiency at which Co is used by ruminal microorganisms that produce vitamin B12 is very low, and according to Smith (1987), the amount of Co in the diet converted into vitamin B12 in the rumen ranges between 3–13% of the intake [25,26]. The amount of cobalamin synthesized depends on multiple factors, including the composition of the ration (ratio of forage to concentrate; fiber content) and dry matter intake [20]. However, it has long been recognized that the most important factor for the production of vitamin B12 and analogs synthesized by ruminal bacteria is the concentration of cobalt in the diet [9,17,20]. Without cobalt in the diet, ruminal production of vitamin B12 decreases rapidly (within a few days). However, vitamin B12 stored in the liver of adult ruminants is usually sufficient to last for several months when placed on a cobalt-deficient diet [19].

Ruminants appear to be more sensitive to vitamin B12 deficiency than nonruminants, in large part because they are highly dependent on gluconeogenesis to meet their tissue glucose needs. A decompensation in propionate metabolism at the point where methylmalonyl coenzyme A (CoA) is converted to succinyl-CoA may be a primary problem arising from vitamin B12 deficiency [19].

The purpose of this contribution is to establish the existing nexus between a mineral element, cobalt, with the interpretation of its role in the synthesis of vitamin B12, as well as its participation in animal metabolism, especially in ruminants. First, we will analyze the effect of this element from a physiological point of view, and later, we will interpret the effect of both a possible deficit and an excessive cobalt ingestion. Finally, we want to provide possible diagnostic methods and some management protocols in the contribution of cobalt to the feeding of cattle and sheep.

## 2. Cobalt and Vitamin B12 Essential Functions

Vitamin B12 or cobalamin is an essential part of several enzyme systems that carry out a series of very basic metabolic functions. It is crucial for energy metabolism and for cell replication processes, since it behaves like a coenzyme, catalyzing intramolecular mutations and reactions of transfers of one-carbon groups [7]. Most cobalamins serve as a cofactor for important enzymes; thus, the metabolism of carbohydrates, lipids, amino acids and DNA involve reactions in which vitamin B12 is an essential cofactor [4,10].

### 2.1. Forms of Vitamin B12

**Figure 2:**
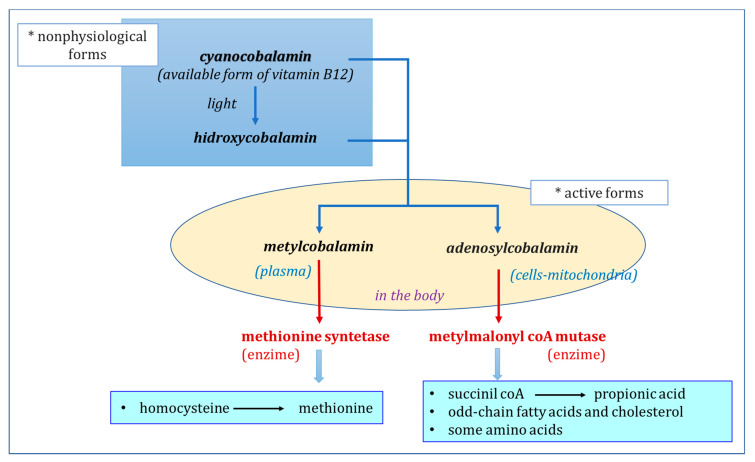
The forms of vitamin B12 and their role in mammalian metabolism. CoA: coenzyme A.

Cyanocobalamin, the generally available form of vitamin B12, and hydroxycobalamin are nonphysiological forms of cobalamin, which are rapidly transformed in the body into methylcobalamin or deoxyadenosylcobalamin (or 5-deoxyadenosylcobalamin), the physiologically active forms or coenzymes of vitamin B12 (Figure 2) [1,9,24,25]. Cyanocobalamin on exposure to light and reducing agents rapidly changes to the form of hydroxycobalamin [10]. Hydroxycobalamin, methylcobalamin and deoxyadenosylcobalamin are chemically more labile than cyanocobalamin (Figure 1 and Figure 2) [1,19,25].

Most of vitamin B12 is found in cells, in mitochondria, in the form of 5′-deoxyadenosylcobalamin, whereas methylcobalamin is the main form of cobalamin in plasma, although small amounts of this coenzyme can be found in cells. Other Co-containing corrinoids, which are not cobalamins, have been detected in plasma and other organs, called cobalamin analogs due to their structural similarity to the vitamin, from which they differ due to alterations in the corrinic nucleus. The biological significance of these cobalamin analogs is not well-known, and although some may be inert, it is suspected that others may have coenzyme activity, be toxic or even inhibit the action of vitamin B12 [10].

### 2.2. Participation in Biochemical Reactions

At least 10 different vitamin B12-dependent metabolic reactions have been identified in microorganisms. However, in mammals, only two important enzyme systems are recognized in which three vitamin B12-dependent enzymes participate: methylmalonyl CoA mutase and leucine mutase, which require adenosylcobalamin as a coenzyme, and methionine synthetase, which is required by the coenzyme methylcobalamin [1,7,10,12,15,16,19,24,27,28,29].

(1) The first, methylmalonyl CoA mutase, is a mitochondrial enzyme, dependent on cobalamin, involved in the metabolism of propionate to succinate. Methylmalonyl-CoA mutase catalyzes the transformation of methylmalonyl-CoA into succinyl-CoA, where 5′adenosylcobalamin functions as a coenzyme of the mutase, allowing the transformation of methylmalonyl CoA, which, in turn, comes from the propionate formed well as a product of ruminal fermentation, of the degradation of odd-chain fatty acids or of some amino acids: valine, isoleucine, methionine and threonine [25,28]. In ruminants, methylmalonyl-CoA plays a unique regulatory role in gluconeogenesis and fatty acid oxidation [30].

**Figure 3:**
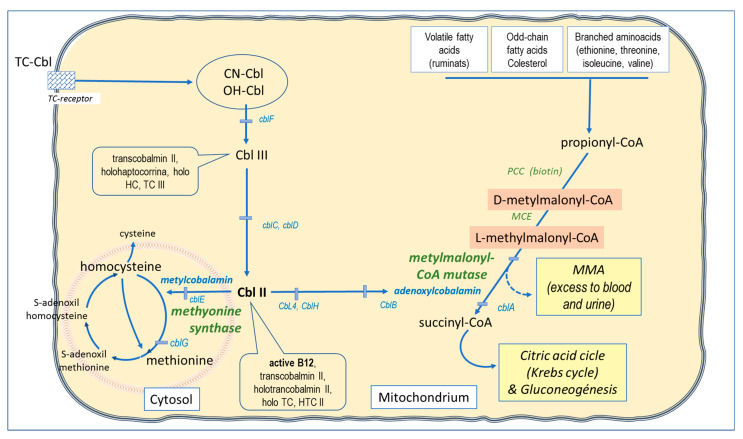
The participation of vitamin B12 in biochemical reactions. Enzymatice systems in which it participates as a coenzyme.

In short, propionate is first transformed into propionyl-CoA; then, through the action of propionyl-CoA carboxylase, a biotin-dependent enzyme, it receives an additional carbon and is converted to methylmalonyl-CoA. Under the action of methylmalonyl-CoA mutase, a vitamin B12-dependent enzyme, the CO- succinyl-CoA (SCoA) group is transferred from one carbon to another in the molecule, forming succinyl-CoA, which can enter the Krebs cycle (Figure 3) [28]. Two molecules of adenosyl cobalamin are required to convert methylmalonyl CoA to succinyl-CoA. In the case of vitamin B12 deficiency, the activity of methylmalonyl CoA mutase is affected; methylmalonyl-CoA accumulates and degrades to methylmalonic acid [14,28,31]. This is a critical reaction for glucose homeostasis in ruminants, because propionic acid is their most important energy source and will be used as a gluconeogenic precursor (Figure 3) [27,28,32,33].

As previously indicated, methylmalonyl-CoA mutase is also an enzyme that participates in the degradation of odd-chain fatty acids and in the metabolic pathways that involve branched-chain amino acids and cholesterol. It catalyzes the conversion of methylmalonyl-CoA to succinyl-CoA, which enters the Krebs cycle for catabolic utilization. The Krebs cycle accepts only two-carbon molecules, so odd-chain fatty acids could not be fully catabolized without this pathway involving the cofactor cobalamin. In the absence of adenosylcobalamin, an accumulation of methylmalonic acid (MMA) occurs as a by-product [7].

(2) Methionine synthetase (or 5-methyl-tetrahydrofolate-homocysteine methyl transferase) is an intracellular, cytosolic enzyme that requires methylcobalamin as a cofactor and that catalyzes the conversion of the amino acid homocysteine to methionine. This reaction links the metabolism of cobalamin and folate (Figure 3) [28,32]. It plays an essential role in the transfer of methyl groups between methyltetrahydrofolate (the methylated form of folic acid) and homocysteine to regenerate methionine and tetrahydrofolate, two essential compounds for the synthesis of S-adenosylmethionine and nucleic acids [14,16,25,27,28,32]. Additionally, methionine synthetase is crucial for nucleic acid synthesis (tetrahydrofolate is a precursor of purine and pyrimidine synthesis) [7].

(3) Another enzyme, leucine mutase (or L-leucine-2,3-aminomutase), participates in the isomerization of L-α-leucine to L-β-leucine. The metabolic importance of this enzyme has not been characterized but has been identified in rat liver and kidneys, sheep and monkey liver and human leukocytes [28].

So far, in dairy cows, the roles of only two vitamin B12-dependent enzymes have been described [28,31]. On the one hand is methionine synthase, described above, which plays an essential role in the transfer of one-carbon units from the methylated form of folic acid to homocysteine [28]. On the other hand is the methylmalonyl CoA mutase enzyme involved in neoglucogenesis, in the passage from propionate to succinate, facilitating its entry into the Krebs cycle (Figure 2 and Figure 3) [28]. In the opinion of Girard and Matte [28], it is likely that this metabolic pathway plays an important role in the energy metabolism of dairy cows.

Cobalt deficiency has been shown to negatively affect the immune function of ewes and calves, leading to increased susceptibility to infection in ewes, with particularly serious consequences for the viability of newborn lambs [9,34]. Even the increase in the synthesis of vitamin B12 by ruminal microbes restored neutrophil function [34,35] and was able to reduce stress [36].

## 3. Cobalt Metabolism: Absorption, Storage and Excretion

### 3.1. Factors that Modify the Production of Vitamin B12

The metabolism of cobalt (and its radioisotopes) has been studied in humans, in laboratory animals, in companion animals and in pets and, especially, as a component of vitamin B12. It is highly probable that the efficacy of the bacterial use of cobalt for the synthesis of cobalamin is influenced by multiple factors. These include the adequate contribution of cobalt [11,15,37], the ingredients and composition of the diet and their effects on fermentation and the composition of the ruminal microbiome [9,15,17,37].

The composition of the diet, and especially the forage-concentrate ratio, play a fundamental role. The synthesis of vitamin B12 is positively associated with the dietary concentrations of neutral detergent fiber (NDF) and acid detergent fiber (ADF) and is negatively correlated with the concentration of starch in the diet [38,39]. Thus, the synthesis of vitamin B12 in the rumen is three times higher in cows that receive a diet high in fiber than in cows that receive a diet high in starch [39].

Other factors, such as initial body reserves, the genetic selection of animals [40,41], the supplementation of different vitamins (alone or in combination) and even the adequacy or deficiency of other nutrients, play an important role in the production and use of vitamin B12, causing variable responses [12,17,22,30,37,40,42].

An important factor is the composition of the ruminal microflora [17]. The microbial synthesis of vitamin B12 is complex due to its structure and the high metabolic cost of its synthesis. Only a few, but phylogenetically diverse, bacteria and archaea are known to produce it [43,44]. By examining the microbiome of the bovine rumen, feces and milk and trying to understand how the ruminal population affected the production and status of vitamin B12, it was determined that the composition of the microbiome of the bovine rumen correlates well with the concentration of vitamin B12. Therefore, manipulating the rumen microbiota can be a good technique to improve the production of this important vitamin [37]. Although Brito et al. reported that only four species of 21 studied species of microorganisms present in the rumen (*Selenomonas ruminantium, Megasphaera elsdenii, Butyrivibrio fibrisolvens* and an unnamed species) were able to synthesize corrinoids, with the first two species producing the highest amounts of vitamin B12 and analogs [17,21]. Although not exactly known, it appears that up to 80% of a dietary supplement of cyanocobalamin, the synthetic form of vitamin B12, is catabolized in the rumen [11,17].

However, neither feed differences nor heritability nor herd management practices fully explain the variations of vitamin B12 in milk [40,41,45], and given that vitamin B12 is synthesized exclusively by bacteria and archaea, it is likely that the composition of the bovine microbiota plays a determining role in the variability observed in the production of vitamin B12 in the rumen [37].

### 3.2. Absorption of Cobalt and Vitamin B12

Most mammals, including humans, do not synthesize B12 vitamins but, rather, acquire them preformed through the diet or through the absorption of vitamin B12 produced by intestinal or ruminal bacteria [1,7]. These vitamins, specific micronutrients for humans and animals, are absorbed by a complicated process [1].

The gastrointestinal absorption of cobalt salts varies according to the species, the compound administered, the dose, the presence of other substances and even the time since ingestion of the food [1]. For example, in rodents, less than 5% of an oral dose of cobalt oxide is absorbed, compared to 30% of an oral dose of cobalt chloride [1]. Increasing the cobalt dose does not tend to cause a significant build-up, as it results in less absorption. In humans, approximately 80% of ingested cobalt is excreted in urine and approximately 15% in feces [1]. Cobalt ions are actively absorbed through the lumen of the small intestine by a divalent cation transporter that also transports other divalent cations, such as iron, copper and zinc, and operates under the influence of vitamin D [1].

Plants and plant products contain practically no cobalamin; therefore, in ruminant herbivores, ruminal microorganisms are the only natural source of vitamin B12. Although cobalt is required to synthesize vitamin B12, McDowell [12] indicates that the conversion of Co from the diet to vitamin B12 is generally very low, since, in ruminants, Co is absorbed with some difficulty. Approximately 3% of ingested cobalt is converted to vitamin B12 in the rumen, and of the vitamin B12 produced, only 1% to 3% is absorbed [12]. Thus, after the oral or intraruminal administration of labeled radioactive cobalt to sheep and cattle, between 84% and 98% appears in the feces between five and 14 days later [19]. The low absorption can be explained by the rapid capture of this element by ruminal microorganisms.

The ruminal flora is capable of producing all the vitamin B12 required by ruminants, as long as there is sufficient cobalt available in the diet [19]. Therefore, the cobalt content in the diet is the limiting factor, and a deficiency of dietary cobalt easily induces B12 deficiency in cattle and sheep [33]. The amounts of the vitamin in the rumen content are between 12 to 663 times those available in food [21].

Ruminants have stomachs that consist of four chambers, with various microorganisms, including those bacteria capable of synthesizing vitamin B12. The synthesized cobalamin is absorbed in the intestine, transferred to the blood and stored in the liver and muscles, or it is secreted in milk [33]. However, there is little information about the mechanisms of the absorption of vitamins in ruminants, so, in most situations, those known for monogastrics are extrapolated.

It is known that, in single-stomach species, while folic acid is efficiently absorbed in the distal duodenum and jejunum (more than 50% of the amount ingested, either by an active saturable process or by a passive nonsaturable process—diffusion), vitamin B12 is absorbed in the terminal ileum with the participation of a binding protein called the intrinsic factor [22]. As previously indicated in monogastrics, the absorption of B12 depends on the synthesis of a glycoprotein that is the intrinsic factor and on a healthy ileal mucosa for the binding and transport of the B12-intrinsic factor complex [46]. Cobalamin, under normal conditions, is bound to dietary proteins. The oral mucosa and salivary glands secrete a glycoprotein called R protein (R-binder = transcobalamin I = haptocorrin, HC). In monogastrics and in the stomach, CBL is released from food proteins under the action of pepsin and at acidic pH. After that, it binds to haptocorrin (HC), forming the haptocorrin-cobalamin complex and protecting them from gastric degradation, thanks to the glycosylated structure that is resistant to low pH [7,46,47]. Later, in the intestines, the haptocorrin-cobalamin complex is degraded, releasing cobalamins to bind to the “intrinsic factor” (IF), which is a glycoprotein of essentially pancreatic origin in dogs and of gastric origin in humans, forming the complex cobalamin-gastric intrinsic factor (GIF). The intrinsic factor protects cobalamin from intestinal degradation and prepares it for absorption by endocytosis in an elective manner in the distal small intestine, where there are specific receptors for the “intrinsic factor-cobalamin” complex. This receptor (cubam) is located on the brush border of the ileum. After leaving the ileal cells, cobalamin binds to transcobalamin (TC) and is transported through the blood. Only TC bound to CBL can enter cells for metabolic utilization [1,7,32,46,47,48].

In cows, the intrinsic factor has been detected; therefore, it is likely that intestinal absorption is similar to that described for monogastric animals, since the binding of intrinsic factor-cobalamin with specific receptors at the ileum level is an essential step for the absorption of the vitamin [11].

The biphasic pattern of cobalamin absorption could be explained by the presence of two different transport mechanisms [22]. Passive absorption, which is a simple diffusion through the walls of the gastrointestinal tract when high doses of the vitamin are present, could explain the increased flow from the ruminal vein and the low efficiency of the process, approximately 1% of the amount present [22,49]. It could also explain the increased flow of vitamin B12 through the portal drainage during the first hours after supplementation. A second increase in the flow of vitamin B12 through the portal supply occurs 20–24 h after dietary supplementation, which is consistent with an active saturable transport process. An efficient but slow process demonstrated in the ileum of some species involves the binding of vitamin B12, the intrinsic factor and transcobalamin II [22,49]. It has been calculated that, in sheep fed 500 mg of cobalamin for four months, 1–3% of the amount supplied is absorbed [22].

The differences observed in the absorption of vitamin B12 could be related to the species, but it is more likely that they are due to the reabsorption of vitamin B12 secreted in the bile and reabsorbed in the small intestine, with recycling through the enterohepatic cycle, which increases with the duration of the supplementation [22].

Girard and Rémond [50] pointed out that Rérat et al. (1958) failed to demonstrate the absorption of vitamin B12 through the rumen wall in fed ewes, but when they infused a vitamin solution into the empty rumen, vitamin B12 reached the blood circulation through the rumen wall. It was even found that the rumen wall could retain the vitamin and subsequently release it, either into the blood or into the ruminal cavity itself [50]. Additionally, Smith and Marston, in 1970, were unable to detect the absorption of vitamin B12 from the rumen of sheep, but they also observed an accumulation of vitamin B12 in its wall [50].

These same researchers conducted a series of complex tests designed to elucidate the fate of vitamin B12, and also folic acid, through the ovine gastrointestinal tract, measuring the flux of vitamins in different parts of the gastrointestinal tract. To do this, they injected concentrated solutions of these two vitamins into the rumen or cecum of ovine. Initially, in three wethers equipped with ruminal and caecal cannulas, and with catheters in the right ruminal and caecal veins and in the mesenteric artery, blood samples were collected after injection into the rumen of 9 μmol of folic acid and vitamin B12/kg body weight (BW) and after applying half of this dose in the cecum, verifying that there was a net release of these vitamins from the rumen shortly after the injection [50]. Subsequently, in four wethers equipped with a ruminal cannula and catheters in the right ruminal, mesenteric and portal veins, blood samples were taken after an intraruminal injection of 0.32 g of folic acid and 0.98 g of vitamin B12. Finally, they took blood samples from two wethers after an intraruminal injection of 0.32 g of folic acid. They concluded that, in ovine, the main site of the absorption of folic acid is the proximal intestine, while the absorption of vitamin B12 occurs in a more distal part of the small intestine, which coincides with the findings indicated for monogastrics [50].

The apparent values of intestinal absorption differ greatly between vitamins, although losses in the rumen are extensive, especially when food is supplemented. Thus, in steers, the ruminal microflora extensively destroys folic acid and vitamin B12 supplemented with the diet [22,23], and the destruction of vitamin B12 can reach 62.9% in lactating Holstein cows equipped with ruminal, duodenal and ileal cannulas [22]. Girard et al. [11] reported several experiments where approximately 5% of the vitamin B12 produced in the rumen of sheep was absorbed in the small intestine, while this efficiency decreased from 1% to 3% when a dietary supplement of cobalamin was added and with the values of intestinal disappearance in sheep apparently very variable (from 1% to 35%). In cows, 45% of the amount of CBL, which reaches a cannula placed in the duodenum, disappeared, measured with an ileal cannula, whereas, in cows fed a 500-mg cyanocobalamin supplement, only 24.5% of this amount seemed to be absorbed in the small intestine. This efficiency is similar (48%) to that found in steers [23]. In the opinion of Santschi et al. [51], there was almost no disappearance of vitamins (except for nicotinamide and folate) when multivitamin supplements (including vitamin B12) were infused post-ruminally in dairy cattle fed a mixed ration.

In summary, in cows, vitamin B12 after synthesis in the rumen is absorbed in the ileum [22]. In the small intestine, before reaching the ileum, vitamin B12 is bound to the intrinsic factor (IF), produced by the parietal cells of the stomach, and this IF-vitamin B12 complex is bound to a receptor that is expressed in ileal enterocytes and, therefore, absorbed into enterocytes [22,52]. Within these, the IF is degraded, and vitamin B12 is released into the bloodstream from the basolateral side. In blood, transcobalamin binds to vitamin B12 and is responsible for the transport of the vitamin to the tissues [37,52].

In humans, mammary epithelial cells have been shown to have a high affinity for transcobalamin-bound vitamin B12 [53]. The CD320 transmembrane receptor is expressed in mammary epithelial cells and shows a high affinity for the transcobalamin-vitamin B12 complex [53]. After endocytosis by mammary epithelial cells, transcobalamin is degraded in the cell, and the free vitamin is transported to the milk [54]. The same transport proteins that are active in this process in humans have also been found in cattle, and therefore, the process is believed to be similar in both species [37,41]. In dairy cows, the absorption of vitamin B12 by the mammary gland, although closely related to the concentration of the vitamin in the mammary artery, represents only 5.5% of the plasma concentration. Furthermore, the absorption of the vitamin by the mammary gland is 17% greater than the amount secreted in milk [37,55].

### 3.3. Transport and Site of Performance

Cobalt is extensively bound to serum albumin, with 5–12% of the cobalt in human serum [1]. When vitamin B12 enters the portal blood, it no longer binds to the intrinsic factor but to specific transport proteins called transcobalamins, responsible for carrying it into cells [12,48]. Three binding proteins have been identified in normal human serum and are designated as transcobalamin I, II and III. Transcobalamines are synthesized in various tissues, including the intestinal mucosa and the liver, and have been shown to deliver B12 to various tissues, including the liver, kidneys, spleen, heart, lungs and small intestine [12]. TCII is secreted by vascular endothelial cells [7].

The mechanism is not well-known, but it is known that, after leaving the parietal cells of the ileum, cobalamin passes into the blood by binding to two proteins with different affinities for cobalamin, haptocorrin (HC) and transcobalamin (TC) [7]. Already in the blood, cobalamin can be linked to a glycoprotein transporter, haptocorrin (HC), forming holohaptocorrin (holoHC; also called transcobalamin III), or to a non-glycoprotein transporter called transcobalamin II (TCII) to form holotranscobalamin II (holoTC or HTCII), which is the bioavailable circulating fraction of the vitamin [3,7,48,56]. Only holotranscobalamin II (also called active B12) can enter cells for metabolic utilization (Figure 3) [47,48,56]. This holotranscobalamin II is absorbed by the cell through a transcobalamin receptor, also called CD320, and recently, a soluble form of CD320 (sCD320) has been detected in human serum, urine and cerebrospinal fluid [48].

Haptocorrin is bound to approximately 70% of the total serum cobalamins, but the role of this protein in the transport and metabolism of cobalamin is unclear [48]. Only 20% of the total serum vitamin B12 that is bound to transcobalamin (TC) is available for use by tissues [8]. It is important to note that holohaptocorrin represents 80–94% of endogenous vitamin B12 in plasma, while holotranscobalamin, on the other hand, can represent between 6–20% of vitamin B12 or up to 30% [48]. A small amount of circulating cobalamin is bound to transcobalamin I and is, therefore, not available for cell uptake [32]. Transcobalamin II seems to be mainly related to the transport of vitamin B12, while transcobalamin I is involved in its storage [12]. The cobalamin-transcobalamin complex is hydrolyzed in the cell lysosome. Subsequently, cobalamin is transferred to the cytosol, where it is converted to methylcobalamin, the active form, and to the mitochondria, where it is converted to adenosylcobalamin (5′-deoxyadenosylcobalamin) [3,48].

In fact, reverse transport to the liver is believed to exist, because HC receptors are characterized only on hepatocyte membranes [57]. However, active molecules captured by reverse transport could be recovered by enterohepatic circulation [7].

### 3.4. Storage

After absorption into the portal circulation, both vitamin B12 and folic acid flow to the liver. There, part of the folates is used by liver cells, while another part is methylated and released into the bloodstream for later use by peripheral tissues. In contrast, vitamin B12 and other water-soluble vitamins are stored in significant amounts in the body, and up to approximately 60% of the total body stores are stored in the liver [9,12,22].

The tissue reserves of vitamin B12 in cattle, as in humans, can be a thousand times greater than the daily needs [9]. These reserves of vitamin B12 in the liver of adult ruminants are usually sufficient to last for several months, which is not the case with rumen microorganisms. Thus, after a few days of a change in diet to a cobalt-deficient diet, succinate concentrations increase in the rumen, either as a result of the inability of rumen microbes to convert succinate to propionate or due a change in ruminal bacteria populations towards the production of succinate instead of propionate [19]. Therefore, without cobalt in the diet or with deficient diets, the production of vitamin B12 in the rumen decreases rapidly [19]. In contrast, young animals are more sensitive to dietary cobalt deficiency, due to the fact that they have less reserves of vitamin B12 in the liver [19]. In the opinion of Weiss [5], the concentrations of B12 in the liver reflect, in an important way, the ingestion of Co.

In the liver, and in most of the animals that have been studied, the average amount of vitamin B12 is around 1.5 mg [12]. Other organs, such as the kidneys, heart, spleen and brain, also store vitamin B12 and contain between 20 and 30 µg [9,12]. Plasma, liver and kidney concentrations are positively correlated [9], and these values decrease when cattle are moved to a lower level of nutrition and vice versa [9].

The liver of nonpregnant mature sheep is capable of retaining up to 16.5% of a dose of cyanocobalamin (with ^58^Co) administered by subcutaneous injection [22], while, in lactating dairy cows, the liver extracts up to 46% of the vitamin B12 that reaches the portal blood [22]. In the opinion of Girard et al. [22], this difference depends on the species studied. Vitamin B12 is an essential coenzyme for one of the main metabolic reactions, for the entry of propionate into the Krebs cycle [12,22]. While, in sheep, the activity of the enzymes involved in propionate metabolism is similar in the liver and in the ruminal epithelium, in cattle, their activity in the liver is three to four times greater than in the epithelium. Therefore, it is likely that an increased propionate metabolism increases the demand for coenzymes and among them, vitamin B12, which is involved in these reactions. The physiological state of the animals could also be influenced, the demand being higher in lactating cows than in nonpregnant ewes [22].

Both plasma and liver concentrations of B12, as well as folates, increased when the ration was supplemented [58]. The addition of folic acid and vitamin B12 increased, respectively, the concentrations of folate and vitamin B12, both in milk and in the liver [58]; however, the increase in plasma folates and plasma B12 in the liver was lower for those cows fed the two vitamins together [30]. Additionally, intramuscular injections of vitamin B12 caused an increase in the liver concentration of vitamin B12 [38,59].

### 3.5. Distribution Throughout the Body

Cobalt levels in animals are very low compared to other essential metals. In most of the reviewed publications, the concentration of vitamin B12 in blood or plasma was reported, and therefore, although the value of cobalt was not reported, it was directly related to the blood value of the vitamin [3].

In most tissues, cobalt is found in a concentration lower than 0.2 mg/kg. It is not accumulated in any particular organ or tissue, although the liver, heart and bone contain the highest concentrations [3,60]; although, according to Simonsen et al. [61], cobalt accumulates mainly in the liver, kidneys, pancreas and heart, and the relative content in the skeleton and skeletal muscle increases with time after continued cobalt administration. Neither is it accumulated in the fetus, but the maternal cobalt concentration has a significant impact on the level of the mineral in newborns [3]. However, cobalt accumulation in the body has not been proven with the age of the animals [60]. According to Herdt and Hoff [4], vitamin B12 is stored in the liver in such a way that, when the reserves are full, they are normally enough to satisfy all the animal’s needs for periods of more than a year.

In summary, the level of cobalt in the liver and kidneys of adults can be influenced by deficiency or by its supplementation; it is normally associated with vitamin B12 or cobalamin, and when a cobalt deficiency occurs, vitamin B12 also decreases [3].

### 3.6. Elimination and Excretion

The excretion of absorbed vitamin B12 is through the urinary, biliary and fecal routes [7,12,32], and its excretion has even been reported through sweat [3,61]. In general, most of the absorbed cobalt is eliminated through the urine. This elimination occurs quickly; therefore, almost all of the absorbed cobalt (80–90%) has a biological half-life of several days, measured by the excretion of radioactive cobalt after accidental inhalation, but a small amount of the initial dose may even have several years of a half-life [60].

There is a reuptake system in the proximal tubule of the kidneys that recovers the vitamin through a 600-kDa protein called megalin or the low-density lipoprotein receptor-related protein. Megalin has a high specificity for the transcobalamin II-cobalamin complex, mediating the renal reabsorption and retention of cobalamin, which justifies its role in the selection and elimination of active forms through urine [7,32]. López-Alonso [62] points out that, for some essential trace elements, and, among them, Co, homeostatic control is mainly mediated by urinary excretion. After absorption, and as homeostatic elimination from the body is activated, trace elements are introduced into the body in a manner proportional to the intake, which explains why body fluids and tissue contents of these elements generally continue to increase when dietary supplies exceed the needs.

Cobalamin is also excreted via the bile (attached to haptocorrin), and a large amount of this vitamin is conserved through enterohepatic recirculation [32]. Body stores of cobalamin in dogs far exceed the amount of cobalamin that is normally lost through the intestinal tract [32]. In human feces, it has been found that cobalamin, together with cobalamin analogs, represent more than 98% of the total ingested, although most of the ingested cobalamin seems to be converted into cobalamin analogs [63].

In ruminants, and especially in females, it is important to consider excretion through milk. Cow’s milk contains approximately 0.5 µg/L of vitamin B12, while colostrum is four to 10 times more than milk [3]. It has been shown that the composition and management of the diet in dairy herds can affect the concentration of vitamin B12 in milk, mainly because the ruminal synthesis of vitamin B12 is affected by the diet [40,64,65] and because the vitamin is partially secreted by milk [66]. Furthermore, the variability in the concentration of vitamin B12 between cows could be partly explained by genetic factors [40,41]. Even dietary supplementation with folic acid and vitamin B12, alone or jointly, is capable of modifying folate and vitamin B12 concentrations in milk [58].

## 4. Interactions with other Nutrients

It is especially relevant to consider the multiple interactions between different minerals; thus, and by way of example, it can be observed that cobalt interacts with copper, iron, iodine and zinc, according to the so-called Mulder wheel or scheme [67,68,69].

Co interferes with the absorption of iron (Fe) and vice versa. In the rumen, iron interacts with cobalt, such that iron deficiency improves cobalt absorption [12]. Likewise, cobalt and high iron can reduce Se absorption [70]. Increases in iron levels can depress cobalt absorption, possibly because both elements can use a common transporter in the intestinal mucosa [3]. Cobalt ions are actively absorbed through the lumen of the small intestine by a divalent cation transporter that also transports other divalent cations, such as copper and zinc, and which operates under the influence of vitamin D [1,70]. 

In children, clinical manifestations of low cobalamin levels can begin from birth and extend beyond weaning, because these children whose mothers have a low intake during pregnancy and lactation (fed macrobiotic, vegan or low cobalamin diets) have low cobalamin stores and are likely to continue with insufficient intake after weaning [71]. In addition to lower birth weights, a marked decrease in heights, weights and other physical measures were observed from six months onwards, which showed that children with deficiencies can manifest a delay in development. Rickets in children has been associated with these diets in early life and could be caused by deficiencies of relevant nutrients such as vitamin D and calcium [71]. Lower serum cobalamin and higher MMA have been reported in children on macrobiotic diets, associated with lower bone density and mineral contents [72]. In chickens, it has been proven that vitamin D increases the absorption of cobalt and iron in the presence of low levels of calcium [70].

Co can replace Zn in the carboxypeptidase enzyme and replace part of Zn in the alkaline phosphatase enzyme. It has been cited that Co supplementation in pigs can prevent injuries associated with Zn deficiencies [3]. Additionally, the administration of Co increases the urinary excretion of Cu and the concentration of Zn in the liver [70].

It has also been reported that Co can interact with various amino acids. When 0.5% to 1% methionine was administered to pigs, toxicosis caused by feeding 600 mg of Co/kg was alleviated [3]. Supplementation with 500 mg of methionine, intravenously, in calves, when administered before an injection of 50 to 75 mg of Co, prevents or reduces the severity of signs of toxicosis of this element [3].

## 5. Requirements in Ruminants

### 5.1. General Considerations

As we have indicated previously, the needs for vitamin B12 in ruminant diets are closely related to the requirements for cobalt, since, being a component of the B12 molecule, the cobalt content of the diet is the main limiting factor for the synthesis of vitamin B12 by the ruminal microflora. Since cobalamin is not present in foods of plant origins, the supply of vitamin B12 in ruminants must be guaranteed by a sufficient supply of Co [16]. In general, it has been estimated that rumen microbes need between 0.07 to 0.11 mg cobalt/kg of feed to function efficiently [19,73] or an adequate dietary supply of cobalt (0.07 to 0.2 mg/kg) [12]. Ruminant animals have the ability to synthesize vitamin B12, provided they receive an adequate dietary supply of cobalt and have a normally functioning rumen [12]. However, the proportion of cobalt in the diet used for these synthetic processes is relatively low in sheep and cows, varying from 3% to 15% [11,17,20], although, according to Girard et al. [11], only 4% of cobalt in the diet was used for the synthesis of CBL.

Ruminants have higher vitamin B12 requirements than nonruminants, presumably due to their participation in propionic acid metabolism [12]. The surprisingly high requirement by ruminants for cobalt arises, in part, from the low efficiency of vitamin B12 production from cobalt by rumen microorganisms and, in part, from the low efficiency of vitamin B12 absorption [12]. It is important to note that young ruminants, which do not have a fully developed rumen, require a dietary source of vitamin B12, and under normal conditions, a rumen would be functional for the synthesis of all B vitamins from six to eight weeks of age [12]. Brewer et al. determined that the dietary requirement for cobalt is 1.2 to 2.4 mg/day for cattle, varying with the weight and metabolic status, with an intake that should not exceed 25 mg/kg of feed based on dry matter [1]. The current requirement for Co is expressed based on the concentration, as we have indicated, of 0.11 mg/kg in the dry matter (DM) diet, instead of in mg of Co/day absorbable. This is done because Co is required mostly, and perhaps only, by ruminal bacteria, and the amount they need depends on the food available in the rumen [5].

The rumen synthesis of vitamin B12 increases with higher concentrations of Co in the diet [74]. Thus, an increase in serum vitamin B12 has been demonstrated when Co supplemented to the diet increased from 0.10 mg/kg to 1.0 mg/kg (DMB) [75,76,77]. The recommended supplementation of Co, in cattle, should be between 0.11 and 0.35 mg/kg, with a maximum value of 10 mg/kg, and showing toxicity characteristics when 30 mg/kg is reached [78].

Although the data on the concentration of Co in foods are limited, the requirement, according to the National Research Council (NRC), is that the total Co and, in most situations, the basal ingredients of the diet are capable of providing an adequate amount of Co [5]. Thus, multiple investigations have been carried out with basal diets containing from 0.2 to 0.4 mg/kg of Co [74,79] to diets with 1 and 2 mg/kg of Co [5,38]. Stangl et al. [80] estimated the Co requirement of cattle and verified that the recommended levels of Co in the diet can be established around 150–200-µg/kg DM, while, with the Co content in the diet, in maximum levels of vitamin B12 were found around 250 mg/kg of DM [80]. In addition, plasma folate did not show any response to the different levels of Co and hemoglobin, and hematocrit responded slightly to the increase of Co in the diet and only decreased in those bovines with diets below 100 µg of Co/kg of DM [80].

However, there is multiple research that shows that the synthesis of vitamin B12 could be restricted even when the diet is adequate in cobalt, since several factors can influence its synthesis and, perhaps, its use [12]. Among these factors, we can point out some, such as seasonal changes, cobalt concentrations in feed, including grasses, grazing habits, animal species, age, the concentrate-forage ratio, the levels of various other nutrients in the diet and even soil contamination [12,74].

In sheep, the efficiency of cobalt utilization decreased as the concentration of cobalt in the diet increased [17]. On the contrary, in cows fed different diets, it has been reported that the efficiency of the use of cobalt for the synthesis of CBL in the rumen is 7.1%, 9.5% and 4.4% for those diets that provide 0.17, 0.29 and 2.5 mg of Co/kg of DM, respectively [11,17,20], being lower than expected by the researchers.

As early as 1953, Ford et al. reported that the relative proportions of vitamin B12 and its analogs produced in the rumen depend not only on the ingested diet but, also, on the composition of the ruminal microflora [17]. According to Brito et al. [17], only four species of microorganisms present in the rumen (*Selenomonas ruminantium, Megasphaera elsdenii, Butyrivibrio fibrisolvens* and an unidentified species), of the 21 studied, could synthesize corrinoids, and also, the first two species produced the largest amounts of vitamin B12 and analogs [17]. According to Franco-López et al. [37], high concentrations of vitamin B12 in the rumen were correlated with increases in the presence of Prevotella, while lower concentrations of vitamin B12 were correlated with the presence of *Bacteroidetes, Ruminiclostridium, Butyrivibrio, Succinivibrionaceae* and *Succinimonas* [37]. For Santschi et al. [51], the apparent ruminal synthesis of biologically active vitamin B12 was higher with a forage:concentrate ratio in the diet of 60:40 than with a diet with a ratio of 40:60. A positive relationship has even been observed between the concentration of vitamin B12 in milk and the fiber content and a negative relationship between the concentration of vitamin B12 in milk and the crude protein content of the diet. All this suggests that the concentration of vitamin B12 in serum, and even in milk, could be modified by the composition of the ration [40]. Legumes, such as alfalfa or clover, are the main sources of cobalt in the natural diet of some ruminants [1].

When cattle are fed diets high in concentrates, there is an increase in serum concentrations of total vitamin B12, but the proportion of cyanocobalamin decreases because a large quantity of analogs are produced. These natural analogs have little or no activity [9,17]. Tiffany and Spears [77] used fattening cattle to show the effect of dietary concentrate on the ruminal synthesis of vitamin B12, with a higher response to Co supplementation when corn was the grain source compared with barley [74]. 

In the concentrations of vitamin B12 in the ruminal content of heifers fed with a mixture of hay and grain, there was no apparent difference when they were fed with pelleted ground hay or with chopped hay, and furthermore, the ruminal content contained significantly more of the vitamin when fed long hay than when fed ground hay, and both were supplied together with corn [21]. It has even been suggested that requirements for cobalt, as well as copper, higher than the NRC recommendations can help the digestibility of poor-quality forages (such as corn crop residues) [12].

The apparent ruminal synthesis of CBL is negatively correlated with the ruminal pH [81], the disappearance of starch or the intake of starch and is positively correlated with the ADF (acid-detergent fiber) or NDF (neutral-detergent fiber) intakes [17]. Apparently, in contradiction with the effects of rumen pH on the CBL concentration in the rumen, Cannizzo et al. [82] observed that the plasma concentrations of total vitamin B12 increased in cows with a ruminal pH lower than 5.6 [17]. This can be important in episodes of subacute ruminal acidosis, which can only show a low productive performance of the dairy animal or fattening steers, and which is characterized by a low ruminal pH (5.5 to 5.0) as a consequence of the low-fiber ration [83]. 

It is important to bear in mind that the increase in the plasma concentration of folates and vitamin B12, measured together with the liver levels of vitamin B12, after the ingestion of vitamin supplements were lower in those cows supplemented with the two vitamins simultaneously than in those cows that received only one of the vitamins, even when similar amounts were given [27,30].

According to Dupleis et al. [40], the genetic selection and composition of the ration could be used to modify the concentration of vitamin B12 in milk. By sampling 399 dairy cows in early lactation (primiparous vs. multiparous; Holstein vs. Jersey) in 15 commercial herds, they verified that, although the concentration of vitamin B12 in milk ranged between 2309 and 3878 pg/mL, neither the parity nor the sampling time affected the concentrations of vitamin B12 in milk, although the concentration of vitamin B12 in milk was highly variable between and within the dairy herds. The heritability (the proportion of variation due to genetic variation) was 0.23 ± 0.20, which suggests that genetic selection would allow modifying the concentration of vitamin B12 in milk. Rutten et al. [41] reported a heritability value of 0.37 for the concentration of vitamin B12 in the milk of first lactation Holstein Friesian cows.

There are a few studies that reported on trace elements in conventional and organic systems that allow us to accurately estimate mineral deficiencies linked to organic management practices. Thus, in New Zealand, the majority of sheep, cattle and deer have a high prevalence of Co deficiency, along with other trace elements (Cu, I and Se), with mineral deficiencies being a challenge for organic agriculture. Mineral deficiencies, especially the trace minerals Se and Co, showed a regional pattern and were considered as one of the main problems on various farms. In Northwestern Spain, organic meat and dairy cattle showed a lower mineral status compared to animals from intensive systems; although the deficiencies of Co, Cu and Se were only observed in beef cattle that did not receive complementary feed, these deficiencies being more frequent in dairy farms, possibly due to their greater need for milk production [62].

### 5.2. Requirements in Dairy Cattle 

Some experiences about supplementations with Co/vitamin B12 in dairy cattle are shown in Table 1. Although the established dietary requirements for cobalt in ruminants are 0.1 to 0.2 mg/kg [24], it has been suggested that the requirement for vitamin B12 in dairy cattle is between 0.34 and 0.68-µg/kg BW [84]. The surprisingly high needs of vitamin B12 in dairy cows is due to the fact that vitamin B12 is essential as a cofactor of methylmalonyl-CoA isomerase, an enzyme necessary for the use of propionic acid, produced in large quantities in high-production ruminants, in addition to the low efficiency of vitamin B12 production from cobalt by ruminal microorganisms and the low efficiency of vitamin B12 absorption [12].

The production of both folate and vitamin B12 by ruminal microflora is generally considered sufficient to avoid deficiency symptoms in ruminants. However, there is no unanimous criterion about milk production in response to an increased supplementation with Co. Thus, parenteral and oral supplements of vitamin B12, as well as folic acid-increased milk production and modified milk composition in dairy cows, suggest that rumen microbial synthesis may not be sufficient to optimize animal performances [22]. In most investigations, when the total Co of the diet (basal plus supplemental) was approximately 1 to 1.3 mg/kg, maximum responses were observed in milk production [80].

However, such production responses require large amounts of vitamin B12 and folic acid in the diet, which are necessary to increase their plasma concentrations to levels similar to those observed after parenteral supplementation [22].

#### 5.2.1. Milk Production and Reproductive Parameters

In this regard, when milk production and reproductive indices were controlled in a herd of cows that were supplemented with a diet with organic forms of minerals and, among them, with cobalt in the form of Co-glucoheptanate, at concentrations of 2.1 mg/kg in the dry period and 1.1 mg/kg during lactation, they verified an increase in milk production (also an increase in urea N in milk and a decrease in the percentage of milk fat, together with the loss of weight and body condition) [85]. However, they did not have a detectable effect on reproductive indices (early follicular dynamics, corpus luteum measurements, embryonic quality, etc.), as well as on liver and luteal levels of supplemented minerals (Zn, Mn, Cu and Co) [85].

On the contrary, in dairy cows, the concentrations of B-12 in the liver continued to increase as the supplemented Co increased in the ration (also in the form of Co glucoheptonate), with values of up to 3.6 mg/kg [38]. However, this elevation of vitamin B12 values in the liver did not translate into any benefit for the health of the cows or for their production. Therefore, detecting high levels of this vitamin in the liver could not have any important significance [5].

A linear increase in milk production was reported in multiparous cows when the Co supplementation increased from 0 to about 1 mg/kg but without any effect in first lactation animals [5]. Older cows tend to have lower concentrations of B12 in their livers, which could explain the parity effect [5].

When it was investigated in lactating dairy cows, to evaluate the influence of an increase in the supply of cobalt as cobalt-sulphate (CoSO_4_ 7H_2_O) on the microbial synthesis of vitamin B12, on the quality of the milk (protein and lactose concentrations), as well as on some ruminal parameters (pH, concentrations of short-chain fatty acids, ammonia concentration and the synthesis of microbial proteins), they verified that an extra supply of cobalt in the diet (0.29-mg compared to 0.17-mg Co/kg DM) had no influence on the cited ruminal parameters, nor on the characteristics of the milk produced [20]. However, the extra addition of cobalt (0.29-mg Co/kg DM) resulted in higher amounts of vitamin B12 in the duodenal chyme (cobalt content in the duodenal chyme) and with considerable individual differences [20]. Neither did extra oral cobalt supplementation in the ration of pregnant dairy cows, although it caused slightly higher cobalamin concentrations in the serum of the cows, result in an increase in the levels of vitamin B12 in the serum of their calves [18]. Perhaps this was due to the fact that the cobalt content in the ration of dairy cows (0.27-mg Co/kg DM and higher than current recommendations) was higher than in control cows (which received 0.13 mg of Co/kg of DM) and that this last value was lower than the current recommendation on Co supplementation, around 0.20-mg Co/kg DM [18,80,87].

Low serum vitamin B12 concentrations have been observed frequently in dairy cows during early lactation [28]. However, Walker and Elliot observed the opposite pattern; serum vitamin B12 increased from four weeks before delivery to 12 weeks of lactation but decreased after 16 weeks of lactation. Though, in this last experiment, the method used was sensitive not only to the biologically active forms of vitamin B12 but, also, to at least two vitamin analogs [28].

Some researches showed that diet could have an important impact on the ruminal synthesis of vitamin B12, in such a way that the concentration of vitamin B12 in milk could be modified by feeding management but only in a limited way [45]. The concentration of vitamin B12 in milk varied widely between herds, from 2861 to 5892 pg/mL [45].

In the opinion of Duplessis et al. [40,45], the dietary factors associated with the concentration of vitamin B12 in milk are capable of explaining only a limited part of the variation, since its concentration was positively related to the percentage of fiber and negatively with starch and with the energy of the diet, and also, the ruminal synthesis of vitamin B12 was positively correlated with the intake of nutrients and the ingested amounts of NDF and starch [64].

It is also possible to observe a negative correlation between the concentration of vitamin B12 in milk and milk yield, including the concentration of lactose, and a positive correlation between the concentration of vitamin B12 and of fat and protein in milk [45]. However, there are a series of individual factors that also determine the values of vitamin B12 in milk, with the third and successive lactations showing a higher concentration of vitamin B12 in milk than first and second calving cows [45].

Serum concentrations of vitamin B12 decreased dramatically in all cows in the antepartum period; therefore, the dietary supplementation with Co (containing 0.15, 0.89 or 1.71 mg/kg of Co, dry matter basis) can increase the ruminal synthesis of vitamin B12, increasing the concentrations of vitamin B12 in the colostrum and milk of those supplemented cows. However, there was no effect of supplementation on the intake of dry matter or the production of milk and milk components, nor did it affect the concentrations of Co in the liver or serum, although the concentration of Co in the milk did increase (0.089, 0.120 and 0.130 g of Co/mL) at 120 days in milk [74].

#### 5.2.2. Joint Supplementation of Vitamin B12 and Folates

Several investigations concluded that the supplementation of vitamin B12 in dairy cows, together with an adequate level of folic acid, appears to improve the efficiency of energy metabolism in early lactation without increasing the DMI (dry matter intake) [17,30,31,42,58,59]. It has even been hypothesized that this increase is probably due to increased gluconeogenesis caused by the combined vitamin supplement [58,59].

Given the metabolic functions of these two vitamins, folates and vitamin B12, it is likely that the demand for both vitamins may be higher in early lactation than in late lactation, due to the enormous energy requirements for milk production. In this regard, it is important to emphasize the dominant role of gluconeogenesis in the production of glucose, a precursor of milk lactose, the preferential use of propionate as a substrate for gluconeogenesis (60% of glucose is derived from propionate in dairy cows), a large amount of propionate absorbed due to diets rich in concentrates administered to high-performance dairy cows to compensate for their negative energy balance after calving, and that, in addition, folates are involved in the synthesis of DNA, RNA and proteins [22].

Therefore, in 15 dairy herds with different production levels (from 8043 to 11,034 kg in 305 days) and with different types of rations (CP (crude protein) = 16.3% to 18.5% DM (dry matter), NDF (neutral detergent fiber) = 25.6% to 37.6% DM and Co = 0.26 to 0.94 mg/kg DM) given weekly injections of folic acid and vitamin B12, increased body condition score (BCS) and decreased the proportion of fat and protein in early lactation, which seems to indicate that those cows that received the vitamin supplement presented a better energy state than the control cows [42].

Higher production and increased plasma glucose were observed in multiparous cows that received a combined dietary supplement of folic acid and vitamin B12, while no effect was observed in cows that received only a vitamin B12 supplement [30]. The joint supplementation seems to increase the use of both vitamins, especially in the extrahepatic tissues, since it has been verified as a greater performance of lactation and dry matter intake similar to those of cows supplemented with folic acid alone but with an increase of plasma glucose and a decrease in liver lipids [30].

The discrepancy in some responses, and especially in milk production, may be related to the status of vitamin B12 in cows. The availability of vitamin B12, originating from microbial synthesis, was estimated from the plasma concentration. Thus, cows with plasma concentrations of vitamin B12 higher than 200 pg/mL showed greater milk production when supplemented with folic acid than cows with concentrations lower than 200 pg/mL. It appears that a lower availability of vitamin B12 could impair the response of cows to folic acid supplementation. Therefore, milk production increased by 12% in multiparous cows in early lactation supplemented with folic acid and vitamin B12, intramuscularly, compared to control cows or cows that only received a folic acid supplement [59].

Dairy cows, in early lactation, could benefit from a higher supply of CBL, even when the dietary supply of cobalt is adequate. In primiparous cows, intramuscular injections of CBL, in addition to increasing blood hemoglobin and decreasing serum methylmalonic acid, increased the concentrations of the vitamin in milk and milk yields (solids, fat and lactose) compared to cows supplemented only with folic acid [31]. On the contrary, in multiparous cows, the combined supplements of folic acid and CBL, administered orally or parenterally, in early lactation increased the yield of milk and milk components by improving the efficiency of the energy metabolism [11,30,59].

Duplessis et al. [58] studied the effects of folic acid and vitamin B12 supplementation on propionate and glucose metabolism. To do this, they assigned 24 multiparous cows to various experimental groups: (1) 0.9% NaCl saline solution, (2) 320 mg of folic acid, (3) 10 mg of vitamin B12 or (4) 320 mg of folic acid and 10 mg of vitamin B12. Intramuscular injections were given weekly from three weeks before the expected delivery date until nine weeks after delivery. The joint supplements of folic acid and vitamin B12 increased, respectively, the concentrations of folate and vitamin B12, both in milk and in the liver. Although the dry matter intake was not affected by the treatments, the milk and lactose yields tended to be lower by 5.0 and 0.25 kg/d, respectively, for those cows that received the folic acid supplement. The plasma concentration of ß-hydroxybutyrate followed the same trend. In contrast, in those cows that received the combined supplement of folic acid and vitamin B12, methylmalonyl-CoA mutase and S-adenosylhomocysteine hydrolase were higher. These results suggest that the folic acid supplement reduced the synthesis of lactose derived from glucose by redirecting glucose towards the mammary gland or towards other tissues, given that the absence of the effect of the treatment on the plasma concentrations of methylmalonic acid—thus, as in the proportion of glucose synthesized from propionate of around 60%—supported the fact that the supply of vitamin B12 was sufficient for normal metabolism in the trial cows [58].

#### 5.2.3. With other Substances

In a herd with a marginal iodine status, it was found that milk production on day 100 of lactation was significantly higher in cows treated by the application of intraruminal slow-release boluses, which provided iodine, selenium and cobalt. They produced, on average, 224 kg more milk than untreated cows, although the authors pointed out that more studies are required in a larger number of herds and in different management systems to clarify what types of herds could benefit from this type of supplementation [86].

### 5.3. Requirements in Feedlot Cattle

In general, growing animals are at greater risk of Co/vitamin B12 deficiency than adults [4,6]. It has even been reported that, in steers, the ruminal microflora extensively destroys both folic acid and vitamin B12 administered in the diet [22,23]. It is also important to remember that the low level of B12 in the plasma of fattening cattle reflects the high content of ruminal production of B12 analogs, not always active, when the cattle are fed diets high in concentrate [9]. Some experiences about supplementations with Co/vitamin B12 in feedlot cattle are shown in Table 2.

In most experiments, the dietary supply of cobalt is always above the current NRC recommendations [24,84], which suggests that the current cobalt requirement estimates of 0.10 [24] and 0.11 mg/kg [84] may be too low, and especially for beef cattle, the Co requirement may be between 0.15 and 0.25 mg/kg [28,74,76,80].

On those occasions in which the ration of growing calves has been supplemented with cobalt, such as cobalt chloride, with much higher doses (1 and 6 mg/kg), it was found that, regardless of the level of cobalt in the diet, there was no significant effect on the digestibility of the tested diet, as well as on the indicator parameters of the productive performance (average cumulative body weight, net gain in body weight or feed efficiency), or even on the balance of nutrients such as nitrogen, calcium and phosphorus [89]. When the diets of 144 cross calves, stressed during transport, were supplemented with vitamin B at levels up to 10 times higher than those recommended for growing pigs, it did not influence the weight gain or feed conversion during the 56-day trial. However, a vitamin supplementation was able to reduce morbidity [23].

In Germany, Simmental bulls supplemented with cobalt, as cobalt sulfate, between 0.07 and 0.69 mg Co/kg showed much lower concentrations of vitamin B12 in plasma and the liver than in bulls fed lower diets. Therefore, cobalt needs of 0.15 and 0.25 mg/kg of ration were estimated, based on the concentrations of B12 in plasma and the liver, respectively, and dietary Co was inversely correlated with the plasma concentrations of homocysteine and MMA [80].

Those bulls fed a control diet containing 0.07 mg of Co/kg had lower gains, feed consumption and carcass weights at slaughter than the cobalt-supplemented animals, estimating that the cobalt requirements are 0.12 mg/kg for maximum growth and 0.16 to 0.18 mg/kg for maximum feed intake [87]. Furthermore, these results suggest that the cobalt requirements for the maximum productive performance of cattle are lower than those necessary to achieve the maximum concentrations of vitamin B12 in plasma and the liver [87].

Stangl et al. [80] observed plasma concentrations of vitamin B12 of approximately 108 pg/mL in growing cattle when they were fed a cobalt-deficient diet and of 271 pg/mL in those fed with a proper cobalt diet. In beef cattle, growing animals showed maximum hepatic Co values when the diets contained 0.22-mg/kg Co, which is approximately double that currently recommended [5].

### 5.4. Requirements in Sheep

The minimum cobalt requirement for sheep was closely adjusted in the late 1930s, primarily by research conducted in New Zealand. Grasslands containing 0.07-mg/kg cobalt in dry weight were found to be at the cobalt requirement limit for sheep and concluded that a total daily intake of 0.07 to 0.08 mg of cobalt would fully meet the needs of the sheep. This amount is provided by a ration containing 0.08 to 0.10-mg/kg cobalt on dry weight [90].

Experiments with sheep suggest an oral requirement for the growth of lambs of about 200 µg/day, approximately 10 times the oral requirement indicated for other species [12], although, in sheep, the dietary supplement of cyanocobalamin is rapidly destroyed in the ruminal cavity [11]. Early attempts to treat cobalt-deficient lambs with vitamin B12 were negative, due to the use of suboptimal amounts of the vitamin. These observations led to the statement that ruminants must have a high requirement for vitamin B12, which is not shared by Smith and Loosli [90].

We have already indicated that, in sheep, the efficiency in the use of cobalt decreased as the concentration of cobalt in the diet increased [17], as well as that a diet high in corn (60% corn) markedly reduced the amount of active B12 in both the rumen and blood [9].

Intramuscular administration of vitamin B12 at the rate of 100 µg per week or 150 µg every two weeks produces a rapid remission of all signs of deficiency in lambs and is as effective as cobalt administered orally at 7 mg/week [12].

Cobalt deficiency has been shown to negatively affect the immune function of ewes and calves, leading to increased susceptibility to infection in ewes and with particularly serious consequences for the viability of newborn lambs [34]. Cobalt supplementation is capable of restoring neutrophil activity in ovine and bovine species, and it has even been noted that the increase of the synthesis of vitamin B12 by ruminal microbes can reduce stress [36].

## 6. Cobalt Deficiency

Human research, although very extensive, has focused almost exclusively on autoimmune phenomena and pernicious anemia. Studies in animals are limited and almost always correspond to clinical findings, due to difficulties in producing an absolute deficiency in experimental animals. Deficiencies related to vitamin B12 in ruminants can occur within months after feeding a cobalt-deficient diet, whereas, in humans, it can take several years and, with a vegetarian diet, produce a vitamin B12 deficiency. Consequently, ruminants could be a useful model for vitamin B12 metabolism [9].

### 6.1. Epidemiology

Clinical cobalt deficiency in ruminants, both mild and severe forms, is well-described and occurs in certain areas throughout the world [4,6,12]. It is generally associated with the grazing of settled grasslands on sandy and well-drained soils [4].

Sheep are more susceptible to cobalt deficiency than cattle [4,6,91]. Vitamin B12 deficiency, although not exclusive, usually occurs in young ruminants [12]. For this reason, and under grazing conditions, lambs are the most sensitive, followed by adult ewes, calves and adult ovines [12].

Grass meadows are more problematic than legumes, since, generally, legume species are much richer in macroelements than grasses growing under comparable conditions. Trace elements and, in particular, I, Cu, Zn, Co and Ni, are also generally higher in legumes than in grasses grown in temperate climates, with Cu and Zn higher in mixed grasses than in pure ones [62].

Fattening diets with a high content of concentrate are also more predisposing to deficiency, because grains are poor sources of cobalt, and the efficiency for the synthesis of vitamin B12 is lower compared to diets with a higher forage content [4]. However, fattening diets are more easily supplemented with cobalt or vitamin B12 than grass-based diets [4].

The soil pH affects the availability of trace minerals to be absorbed by grass species. Leaching over time from rain causes the soil pH to drop, which affects the balance of trace elements in the soil. A pH of 6.5 is considered to be optimal for a soil that contains trace elements in balanced amounts. With pH values lower than 6.5, the availability of Mo and Se are reduced, and the availability of Fe, Mn, Co, Zn and B increases; the opposite occurs with soil pH values above 6.5. Therefore, the pH of those soils with low contents of Mn, Co, Zn and B should not exceed 6.0, since the superposition of soils with low Co contents can result in Co deficiency [62].

The severe form of Co deficiency in sheep and cattle is known as bush-sickness (New Zealand), coast disease (South Australia), wasting disease (W. from Australia), nakumitis (Kenya), pining (Great Britain), lecksucht (Holland and Germany) and Grand Traverse disease (Michigan) and names such as bush disease, debilitating disease, salt sickness or wasting disease [1,3,12,92].

In addition to clinical deficiency, subclinical cobalt deficiency is of great economic importance, because it occurs in very large areas and is difficult to diagnose. Forage has been reported to contain, in many areas of the world, less than 0.1-mg Co/kg DM, which is not sufficient to meet the requirements of ruminants [16].

### 6.2. Clinical Signs

Ruminants grazing on deficient areas or consuming diets containing less than 0.07 to 0.11 mg/kg of dry matter show signs of cobalt deficiency, manifested as a vitamin B12 deficiency [4,6]. Growing animals are at higher risks for the disease than adults, and in mild cases, they can sometimes appear to be caused by parasitism or poor nutrition [4,6].

**Figure 4:**
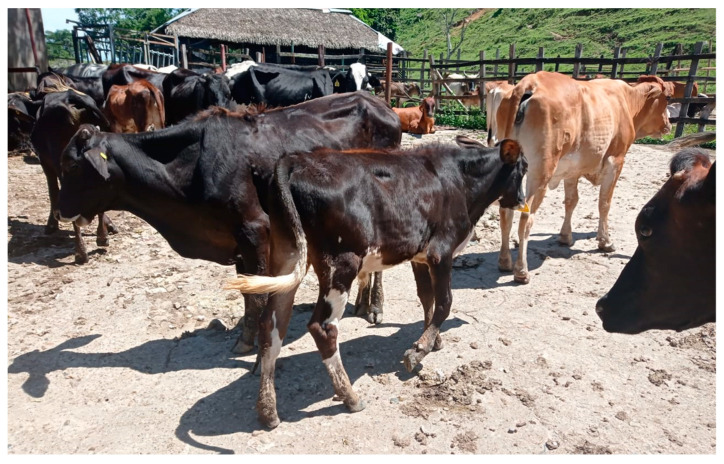
Clinical signs in a cattle herd (photo courtesy of Dr. JJ Bustamante).

**Figure 5:**
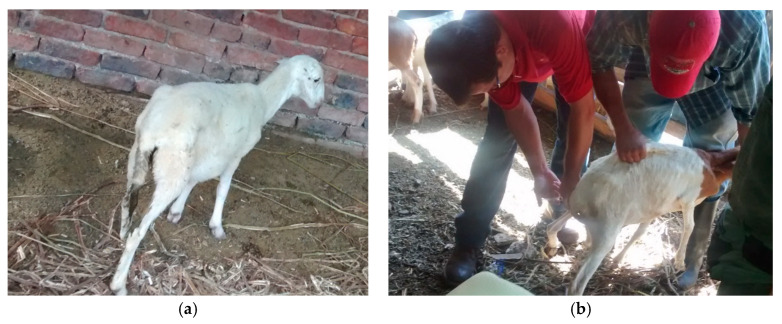
Sheep affected by hypovitaminosis, and parenteral treatment through a supply of vitamin B12 (photos courtesy of Dr. JJ Bustamante). (**a**) Cachectic sheep with signs of diarrhea, (**b**) Parenteral application of vitamin B12.

**Figure 6:**
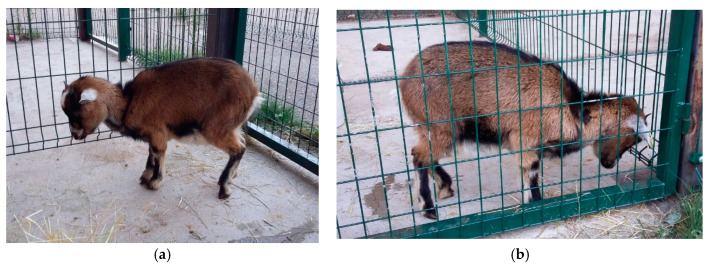
African pygmy goat, with hypovitaminosis B12, secondary to ruminal dysbiosis after repeated ingestion of plastics and other foreign substances (photos from Dr. González-Montaña). (**a**) Young goat, which alternated periods of intense depression with periods of normality. Sometimes with total immobility, which disappeared abruptly, (**b**) The same animal seen from the other side.

Clinically, there is a wide range of symptoms associated with trace element deficiencies, but clinical signs are rarely pathognomonic. The first signs of cobalt deficiency include decreased food intake, reduced growth and weight loss (Figure 4, Figure 5 and Figure 6). More serious signs include diarrhea, fatty liver degeneration, anemia, which provides pale mucous membranes, a decreased resistance to infection as a result of an impaired immune function, impaired reproductive function and even so-called fat cow and downed cow syndromes and, even eventually, death [1,3,4,6,16,19,73,78,92].

Since cobalt is an essential component of vitamin B12, the signs of deficiency are identical to those caused by this vitamin. The symptoms can be explained by the deterioration of the enzymatic functions of cobalamin, which causes, for example, a poor methylation capacity and toxicity of the metabolites, although the effects derived from the alteration of the functions of CBL or a reduced supply, which cause oxidative stress, alterations in the regulation of cytokines or immune responses, are gaining more and more attention [47]. Hepatic lipidosis and megaloblastic, normocytic and normochromic anemia can be attributed to the reduction in the activity of 5’-methyltetrahydrofolate homocysteine-methyl transferase, due to the reduction in the regeneration of methionine and tetrahydrofolate [3]. Inorganic cobalt effects the red blood cell formation, in addition to its role as a component of vitamin B12. In the 1930s, cobalt administration was found to effectively stimulate red blood cell formation [1]. The lower production of tetrahydrofolate will reduce the availability of methyl donors and, therefore, reduce purine biosynthesis and slow cell division, causing megaloblastic anemia [3].

The decrease in the synthesis of methionine can impede the synthesis of choline and, consequently, the transport of hepatic lipids towards the extrahepatic tissues and, finally, can induce hepatic lipidosis, due to a diffuse accumulation of lipids in the hepatocytes [3,93,94,95].

The reduction of the activity of methylmalonyl CoA mutase, a key enzyme in the gluconeogenesis of propionate in ruminants, causes a decrease in blood glucose, increasing pyruvate in plasma and urinary methylmalonate. The elevation of pyruvate and methylmalonate in the blood suppresses the appetite control center in the hypothalamus, causing anorexia, weight loss and even extreme emaciation [3].

Recently, cobalt deficiency has been reported to decrease serum B12 and adversely affect the immune functions of sheep and cattle, with severe consequences particularly for the viability of their offspring [3,35]. Cobalt replacement restores neutrophilic function in both species [3,19].

## 7. Cobalt Toxicosis

Cobalt toxicosis or excess is less common than deficiency, and toxicity is often the result of accidents of excessive supplementation to prevent cases of deficiency [3,4,6,12]. However, for humans, and in excessive amounts, cobalt is classified as a probable carcinogenic compound (group 2B) by the International Agency for Research on Cancer (IARC) [96,97]. In most species, the toxic levels are about 3000 times higher than the requirements [3] or 100 times, according to Dickson and Bond [4,98]. According to McDowell et al. [12], the maximum tolerable amount of cobalt in the diet for ruminants is estimated at 5 mg/kg. Thus, cobalt doses of 4 mg and 1 mg/kg of the body weight can be excessive, causing toxicity for sheep and cattle, respectively [3]. In sheep, the cobalt requirements are 0.1–0.2 mg/kg, and dietary concentrations of 10 mg/kg appear to be safe, with the maximum tolerable level being 10 mg/kg based on the dry matter of the ration. Increasing protein in the diet or administering methionine can be measures to alleviate Co toxicosis, as well as other minerals, such as selenium [3].

Signs of toxicosis include weakness, emaciation, excessive urination, defecation and salivation, shortness of breath, reduced absorption of food, mild polycythemia, increased hemoglobin and packed red cell volume and an elevated Co level in the liver of ruminants [3,12], as well as myocardial degeneration in animals where cobalt injections have been administered [1].

The mechanism of cobalt toxicity is unclear, but some of the effects of cobalt could be related to its high affinity for sulfhydryl groups, which can cause the inhibition of crucial enzymes, such as in mitochondrial respiration [60], to the displacement of the divalent cations in the ionic center of the enzymes activated by metals, to its effect as an antagonist of the Ca^2+^ channel, to the competition with Ca^2+^ for intracellular Ca^2+^-binding proteins and to the generation of reactive oxygen species in cells, which cause oxidative damage to DNA, proteins and lipids [61].

Simonsen et al. [61] pointed out that perhaps the most important effects produced by cobalt could be the activation of the hypoxia-inducible factor (HIF), activated by metals present in almost all animal cells and responsible for a group of sensitive genes to hypoxia, which probably favor tumor development, by promoting the angiogenesis, glucose transport and a series of glycolytic enzymes, which code for the regulation of apoptosis/cell proliferation [61]).

## 8. Diagnostic

### 8.1. General Considerations

There is still no ideal test to measure vitamin B12 deficiency. The cobalt status in ruminants is usually assessed by the direct measurement of blood or tissue concentrations of vitamin B12 or cobalt. In Hunt et al.’s [56] opinion, measurement of the serum cobalamin level remained the preferred option. Although several authors point out that assessing the Co status in ruminants is complicated and unreliable with routine laboratory measurements [4,6,78].

Plasma and the liver levels of B12 have been used extensively to define or determine the cobalt status of the animal, but other interesting options are the measurements of metabolites associated with vitamin B12 deficiency, such as the level of methylmalonic acid (MMA), homocysteine (HCY) or holotranscobalamin (holoTC or active B12) in blood, as well as some hematological variables (hemoglobin, haematocrit, red blood cell count and mean corpuscular volume) [4,48,56,80,99]. Options such as the measurement of methylmalonic acid in the urine, as well as the feed consumption and growth of the animals, and even the response to supplementation therapy, have been proposed as a functional indicator of the animal’s cobalamin level [4,48,80,99].

According to Stangl et al. [80], the determination of hematological variables can only be valuable as a diagnostic indicator in severe Co deficiency, but it is not adequate for estimating the Co requirements. According to Hunt et al. [56], the use of serum holotranscobalamin has an indeterminate “grey area” and needs to be correlated with other laboratory tests. Furthermore, homocysteine tests, being useful, are less specific than methylmalonic acid. Finally, given the variety of techniques and laboratory analyses, the reference ranges are usually established locally, which sometimes causes an inability to define the states of clinical and subclinical deficiency [56].

### 8.2. Cobalt and Vitamin B12 in Blood

The measurement of vitamin B12 in serum is the most common test for evaluating vitamin B12 levels [56]. However, the concentrations of vitamin B12 and cobalt in blood serum, in general, are low, and specialized analyses are needed to measure them [4], although if an automated method is used, the test is available at low cost [56].

It has been cited that interpretation problems may appear, because there are inactive cobalamin analogs in the serum that can interfere with some measurements, and there are multiple serum proteins with which even active cobalamin can bind, further complicating the interpretation of serum values [4]. In addition, in most studies, the correlation between serum cobalt and vitamin B12 concentrations in serum was poor [4]. Additionally, according to Hunt et al. [56], this test measures both serum holohaptocorrin and serum holotranscobalamin and, as such, can mask a true deficiency or misdiagnose a deficient condition.

A decrease in vitamin B12 status is initially signaled by a decrease in holotranscobalamin levels. As tissue reserves of vitamin B12 are used to maintain the metabolic demand, their eventual depletion leads to a deterioration in the performance of those vitamin B12-dependent pathways and, therefore, to an increase in the plasma concentration of homocysteine and methylmalonic acid. Therefore, the concentration of vitamin B12 in the blood decreases more slowly and may not precede a pathological situation, which is due to the fact that the vitamin B12 that is bound to the haptocorrin represents the majority of the vitamin present in the blood. Vitamin B12 bound to haptocorrin is also slowly cleared from the bloodstream, even over several months, which may mask the faster clearance of holotranscobalamin [48].

Serum cobalt concentrations are influenced by the dietary cobalt intake, but this relationship appears variable and inconsistent. This, added to the low proportion of serum cobalt that is present in the combined form of cobalamin, makes the measurement of cobalt in serum of little or no value in the evaluation of the nutritional status of cobalt [4].

Serum cobalt concentrations in adult cattle are between 0.3 and 1.1 ng/mL. Given that vitamin B12 contains 4% cobalt [1,4] or 4.4%, according to Brito et al. [17], cobalamin concentrations represent 0.01 to 0.02 ng/mL of cobalamin in serum; therefore, the total serum cobalt concentrations in cattle are an order of magnitude higher than the expected concentration of cobalamin [4]. For Herdt and Hoff [4], the optimal serum concentrations of vitamin B12 in cattle seem to be in the range of 200 to 400 ng/L or even above, while, for Viglierchio [3], in sheep serum, vitamin B12 is it at a value close to 400 ng/L, and for bovines, it can reach 1000 ng/L. For Aitken [100], serum vitamin B12 levels in sheep should be >370 ng/L (pmol/L or pg/mL).

The serum concentrations of vitamin B12 currently used in diagnosis, at least in Scotland, are as follows: >400 ng/L as adequate, 200–400 ng/L as subclinical (marginal) deficiency and <200 ng/L as clinical (functional) deficiency [101]. Dubeski [9] indicated the following average values and range of vitamin B12 in cattle: of 229 (113–277) ng/L in beef cattle, from 251 (116–396) ng/L in stressed calves, of 286 (150–396) ng/L in dairy cows and dropping to 160 (97–297) ng/L in feedlot. The mean plasma concentration of B12 in the fattening feedlot was 160 pg/mL, which indicated a borderline deficiency, with a very low value of B12, 97 ng/L, in one of the steers [9]. The lower amount plasmatic of vitamin B12 in beef cattle may be related to the production of B12 analogs from a high-concentrate diet [9].

In both species, cobalt supplementation can appreciably increase the serum level of B12 [4]; thus, for example, the serum B12 in cobalt-deficient lambs is 100 to 256 ng/L, compared to 982 at 1613 ng/L in lambs supplemented with cobalt, although it is possible that the analytical method used could have measured both the vitamin B12 and B12 analogs [9]. These same authors reported another investigation in which the serum levels of lambs marginally deficient in cobalt averaged 320 ± 111 ng/L [9]. In contrast, plasma B12 levels in steers averaged 70 ng/L in cobalt-deficient steers fed diets containing 0.03 to 0.04-mg/kg cobalt, compared to 180 ng/L in cobalt-supplemented cattle [9]. However, the estimated Co requirements from the serum vitamin B12 status are usually somewhat higher than with other variables, suggesting that the level of vitamin B12 may increase as the level of Co in the diet exceeds that required for a minimum value of homocysteine and MMA, which, in turn, is necessary for normal growth [80,87].

In most cases, the measurement of vitamin B12 levels is carried out in conjunction with the assessment of serum folic acid [46]. Thus, plasma and the liver concentrations of folates and vitamin B12 increased significantly in animals with supplemented diets [28].

### 8.3. Assessment of MMA in Serum and Urine

Perhaps the most sensitive method to assess the cobalt status in ruminants is through the measurement of methylmalonic acid (MMA) concentrations in serum or urine [4,56]. Greibe [48] pointed to the serum concentration of MMA as a very sensitive and specific method to detect cobalamin deficiency and considered it the gold-standard indicator to assess the status of vitamin B12 [48,102].

Adenosylcobalamin is a cofactor of the enzyme methylmalonyl-CoA mutase that converts methylmalonyl-CoA to succinyl-CoA. The excess of succinyl-CoA becomes MMA [48]. Therefore, the concentration of methylmalonic acid in serum reflects the availability and utilization of adenosylcobalamin by the mitochondria. MMA accumulates when methylmalonyl coenzyme A is not converted to succinate, since this conversion is a cobalamin-dependent process [31,48]. Therefore, the measurement of the plasma methylmalonic acid level has been proposed as a functional indicator of the animal’s cobalamin status [3,48], and high concentrations of MMA indicate a deficiency of cobalamin [4,56], and this persists for several days even after starting supplementation [56]. Girard and Matte [31] pointed out MMA values that evolved from 0.586 (controls) to 0.483 µmol/L in primiparous cows from 25 to 125 days of lactation that were supplemented with weekly injections i.m. (intrasmuscular) of 10 mg of vitamin B12, significantly decreasing the serum concentration of methylmalonic acid while increasing the hemoglobin and packed cell volume. Duplessis et al. [58] reported that the plasma concentration of methylmalonic acid was lower, even in control cows (0.37 µmol/L), than that reported by Girard and Matte [31].

Serum concentrations of vitamin B12 and methylmalonic acid (MMA) have also been used to monitor the evolution of sheep with cobalt (Co) deficiency and their response to supplementation [101]. Although it is true that some results obtained suggest that serum vitamin B12 should not be used in the diagnosis of Co deficiency, at least in individual pregnant ewes, serum concentrations of MMA can be considered a more active and accurate marker of deficiency, and they could be used for this purpose and, especially, to assess the disease prognosis and in subclinical diseases [101].

The use of serum concentrations of MMA, as well as those of homocysteine, as markers for the cobalamin status, although advantageous in most cases, may have certain limitations [48], since they are altered by some factors that should be considered when interpreting laboratory results; such as in people, they increase in the elderly or when there is renal failure [48,56].

Additionally, the appearance of methylmalonic acid in the urine can be used as an indicator of vitamin B12 deficiency, since vitamin B12 deficiency can limit the production of methionine and, thus, limit nitrogen retention [19]. Allen [102], in addition to recognizing serum MMA as a gold-standard indicator, also pointed out that urinary methylmalonic acid (UMMA) was elevated in cobalamin deficiency.

### 8.4. Homocysteine in Blood

Like MMA, homocysteine (HCT) is elevated in the blood of patients with cobalamin deficiency and is used as a functional marker of the cobalamin status [32,48]. Methylcobalamin is a cofactor of methionine synthase, the enzyme that converts homocysteine to methionine, and transfers methyl groups from 5-methyltetrahydrofolate to homocysteine [48]. Therefore, the level of homocysteine in the blood can increase significantly when there is a cobalamin deficiency, as well as in folate deficiency [3]. According to Greibe [48], plasma homocysteine concentrations are more affected by folate deficiency than by cobalamin. In lactating primiparous cows treated with weekly injections i. m. at 10 mg of vitamin B12, the serum concentration of serum homocysteine decreased from 3.50 (in control cows) to 3.42 µmol/L in supplemented cows [31].

There is consensus that significant increases in plasma concentrations of MMA and homocysteine may indicate Co deficiency in cattle, but according to Stangl et al. [80,88], these metabolites are not sensitive enough to detect the optimal Co status in the cattle and, also, their use as markers [80,88], may have limitations in some patients [48]. Hunt et al. [56] reported that plasma homocysteine titration may be helpful but is less specific than methylmalonic acid.

On the contrary, in the opinion of Schwarz et al. [87], homocysteine and MMA, together with the level of vitamin B12 and liver folate, seem to be useful predictors of Co vitamin B12 deficiency and are a valuable tool for assessing Co requirements in cattle. Plasma levels of homocysteine and hepatic folic acid appear to be as sensitive to Co status as food intake and growth [88].

### 8.5. Holotranscobalamin in Blood (HoloTC or Active B-12)

Holotranscobalamin, the metabolically active form of vitamin B12, can be measured by immunoassay [56]. The novelty of using holotranscobalamin as a laboratory marker of vitamin B12 status is based on the fact that the cellular absorption of the vitamin depends on a process of endocytosis mediated by receptors that involve transcobalamin, a plasma protein that transports vitamin B12. Only a minor fraction of circulating cobalamin binds to transcobalamin, and it is this fraction that is known as holotranscobalamin or “active B12” vitamin [48].

A low level of holotranscobalamin is a more reliable marker of the impaired vitamin B12 status than a low level of serum vitamin B12. Furthermore, holotranscobalamin may be an earlier marker to detect vitamin B12 depletion [56]. According to Hunt et al. [56], although this test is being adopted more and more, discrepancies persist on the mode of application and the assignment of cut-off values, which these researchers call the “indeterminate grey area”; therefore, they recommend a second test, such as quantifying methylmalonic acid levels, especially if the result is in the intermediate range.

### 8.6. Cobalt and Vitamin B12 Levels in the Liver

Cobalt concentrations can be measured in serum or tissues. The liver is the tissue of choice to measure both cobalt and vitamin B12, because, although the concentrations are small, it is the place with the highest concentration of cobalt and is the storage site for vitamin B12 [4]. However, there is a variable, and often large, proportion of cobalt in serum or the liver that is not associated with vitamin B12, making the interpretation of cobalt concentrations in serum or the liver difficult [4].

Liver biopsies, to determine B12 concentrations and Co status, in a similar way to how it is done to assess Cu, are invasive methods, sometimes aggressive and are not routinely performed [5]. Bovine liver cobalt concentrations less than 0.1 µg/g (100 µg/kg) of dry liver tissue as cobalt and vitamin B12 deficiency values apply to growing calves and adult bovines but not to cobalt concentrations in fetal and neonatal bovine livers, where cobalt concentrations greater than 0.04 µg/g (40 µg/kg) of dry tissue are probably sufficient [4]. High cobalt concentrations may appear in the liver (mean value of 30 mg Co/kg, with a range of 6–151 mg Co/kg), since, after intravenous administration of cobalt, the liver accumulates approximately 20% of the administered dose of radioactive cobalt chloride [60].

For López-Alonso et al. [103], the cobalt concentration in the liver was 95.4 µg/kg, with values between 43.7 and 152.6 µg/kg, and these researchers also pointed out that, for most of the essential trace elements, the liver was the organ that showed the highest concentrations, having verified both the existence of positive correlations between Co and other trace elements in liver, as well as also antagonism between Co, Mn and Zn [103].

The correlation between liver cobalt and liver vitamin B12 concentrations is only moderate (r2 = −0.42) but is higher when there are low liver cobalt concentrations relative to higher concentrations [104]. In contrast, for Stangl et al. [80], the concentrations of B12 in the liver did reflect the intake of Co. It is assumed that the adequate hepatic concentrations of B12 are between 200–400 nmol/kg on a wet weight basis [80]. For this reason, and using the correlation indicated previously by Mitsioulis et al. [104], concentrations of bovine liver cobalt less than 0.1 µg/g (100 µg/kg) of dry liver tissue are at least suggestive of cobalt and vitamin B12 deficiency in cattle [4].

### 8.7. Cobalt and Vitamin B12 in Milk

There are multiple researches about cobalt levels in the milk of domestic ruminant females. Already, in 1992, Rashed contributed the cobalt values in the milk of cows (9.7 ± 0.25), camels (8.7 ± 0.25), goats (9.6 ± 0, 55), sheep (5.7 ± 13) and buffaloes (5.5 ± 0.30 µg/L), finding the highest concentrations in sheep’s milk than in the rest of the sampled females [105]. Co has even been measured in yak milk, comparing it with cow, sheep and goat milk, and although they indicated that there were clearly very high concentrations of Co, they did not publish the amounts found [106].

According to Coni et al., the differences in the diet may be the main cause of the Co content in cow’s milk, justified by the fact that the variation in the mineral content between the winter and summer feeding showed that there were clearly notable differences in the concentrations (0.010 to 0.079-µg/g dry weight) [107].

It is evident that the dietary composition and management largely determines the status of cobalt in milk; thus, when evaluating the amount of Co in milk from organic farms and conventional farms, it was found that the concentrations were significantly lower in organic milk than in conventional milk (organic farm: 4.77 (4.04–5.26) vs. conventional farm: 4.67 (4.18–5.56) µg/L), and also, the content of organic milk showed a seasonal pattern, possibly related to a higher consumption of concentrated feed in the winter, as well as the ingestion of soil during grazing [108]. According to Miedico et al. [109], the geographic sampling location plays a substantial role in the levels of some metals in milk and, therefore, cobalt levels (ewes: 3.88 (0.540–7.30) and goat: 2.60 (1.27–4.20) µg/L). On the contrary, they hardly gave importance to other factors, such as the species sampled and the time of year [109].

The concentration of vitamin B12 found in the milk of ruminants such as sheep (7.1 µg/kg of milk), cow (0.35 µg/100 g of milk) and goat (0.6 µg/kg of milk) were higher than those found in human milk (0.4 µg/kg of milk) [33]. Nohr and Biesalski [110] reported in 2009 that the amount of cobalamin present in the milk of different ruminants, including buffalo, cow, goat or sheep, was always below 1 µg/100 g, while, a few years later, in 2016, proposed values of 0.51 in sheep’s milk, 0.42 in cow, 0.30 in buffalo and 0.07 in goat, always in µg/kg [111].

Several researchers have verified a great individual variability in the concentration of vitamin B12 in bovine milk, ranging from less than 1.0 to 12.9 ng/mL [40,41,112]. Duplessis et al. [112] cited average concentrations of vitamin B12 in milk of 3.8 and 3.2 ± 1.4 ng/mL, respectively, at 11 and 39 days in milk (DIM). When they measured the concentration of vitamin B12 in the milk of 399 early lactating cows, it ranged from 1.213 to 6.755 ng/mL [40] and from 2.861 to 5.892 ng/mL [45], and when taking into account the number of lactation, the concentration of vitamin B12 in the milk averaged 3.809 ± 0.080 ng/mL, 4.178 ± 0.079 pg/mL and 4.399 ± 0.077 ng/mL for the first, second and third for higher lactation cows [45].

In recent years, several studies showed that diet could have an important impact on the ruminal synthesis of vitamin B12, observing that the concentration of vitamin B12 in the milk of cows in early lactation could be modified by feeding management [39,40,45,64] but only to a limited degree [45]. Therefore, nutrition could help to optimize the concentration of vitamin B12 in milk; thus, the amount of fiber in the ration was positively correlated, while the amount of starch in the diet was negatively related to the concentration of vitamin B12 in milk [45]. Most of the variation seems to be related to the individual attributes of each cow [45].

In the opinion of Rutten et al. [41], this variability between dairy cows could be partially explained by the genotype of the animal [41]. Therefore, the concentration of B12 in the milk of Holstein cows seems to be generally higher than those of the milk of Jersey cows [33], although other factors such as cow type, reproductive status, milking time and feeding management are capable of modifying the B12 concentration of bovine milk [33,37,40,41]. Additionally, the dietary supplementation with Co tended to cause an increase in the concentration of vitamin B12 in colostrum and milk sampled at 120 days in lactation (0.130 g of Co/mL) [74].

Greibe [48] believed that the cobalamin content in milk (4.0–5.0 µg/kg) was moderate, although its bioavailability was considered high [113]. On the contrary, for Franco-López et al. [37] and Bueno-Dalto et al. [114], dairy products are an ideal source of vitamin B12, since it is present naturally and abundantly in cow’s milk [113]. In addition, it is a very stable molecule, which is not destroyed by daylight or prolonged storage [37], while radiation or heat treatments cause variable losses [37,110,111], being pasteurization and UHT (Ultra High Temperature) the ones that cause the least effects [110,111].

### 8.8. Other Analyses

Other variables capable of reflecting vitamin B12 deficiency include complete blood count (packed cell volume and hemoglobin, reticulocyte count, differential leukocyte count, etc.) and lactate dehydrogenase [56].

Although macrocytosis is the most common finding to verify a vitamin B12 deficiency, a bone marrow biopsy is rarely necessary but may be indicated in selective cases where the diagnosis by other laboratory tests is not clear [56].

## 9. Prevention and Treatment

### 9.1. Basic Premises

It is important to take into account that cobalt is needed to synthesize B12, that it has been shown that the conversion of Co in the diet to vitamin B12 is generally very low and that, in general, it is necessary to supplement the diet with Co [36].

It is probable that factors such as the initial body reserves, the composition of the diet, especially the forage-concentrate ratio, the genetic selection of the animals, the supplementation of different vitamins (alone or in combination), the composition of the ruminal microbiome and even the adequacy or deficiency of other nutrients play an important role in the production and use of vitamin B12, causing variable responses [12,17,22,30,37,40,41]. Although not exactly known, it appears that up to 80% of a dietary supplement of cyanocobalamin, the synthetic form of vitamin B12, was catabolized in the rumen [11,17].

The commercial synthesis of vitamin B12 is highly complicated from a technical point of view, with around 70 steps, which makes any industrial production by chemical methods too difficult and expensive. Therefore, currently, vitamin B12 is produced exclusively through biosynthetic fermentation processes, using selected and genetically optimized microorganisms [15].

It is generally accepted that the cobalt requirement in ruminants is 0.1 mg/kg [115], but recently, it was hypothesized that it could possibly be increased in ruminants fed predominantly cereal crop residues to achieve optimal fermentation of the rumen [80]. In addition to synthesis, supplemental Co can also affect the rumen environment and increase the fiber digestion and energy production [36]. It is estimated that the rumen microbiota needs 0.11% cobalt in the ration to function efficiently [19], although there are hypotheses that cobalt supplementation above this minimum requirement may be beneficial for ruminal fermentation, by increasing the growth and activity of microorganisms [89].

The response to supplementation and a diet containing less than 0.08-mg Co/kg DM are useful to diagnose a Co deficiency in ruminants. According to Viglierchio [3], the first notable response to Co feeding is an increase in appetite, followed by an increase in the concentration of hemoglobin in the blood.

### 9.2. Alternatives to Provide Cobalt/Vitamin B12

Cobalt deficiency in grazing ruminants can be prevented by the oral administration of cobalt through mineral supplements, and especially, during critical periods, cobalt supplementation should be continuous [12]. In contrast, ruminants that consume concentrates are more likely to receive adequate amounts of dietary cobalt than grazing cattle [12].

This use of B12 supplementation may be justified under certain conditions in which stress, disease or parasites decrease the food intake, deteriorate ruminal function and/or reduce intestinal absorption. Thus, animals sometimes receive injections of vitamin B12, along with other vitamins, as an insurance for a rapid adaptation to new feeding and management regimes [6,12,94].

In the opinion of Girard et al. [11], the use of an oral cyanocobalamin supplement was not an efficient means to increase the supply of vitamin B12 to cows, because only 4% of cobalt in the diet was used for the synthesis of CBL, and 80% of the supplement disappeared before reaching a cannula placed in the duodenum.

In extensive or grazing systems, the ingestion of soil can contribute significantly to the exposure of livestock to trace minerals. This is because the concentrations of minerals, including cobalt, in the soil are one or two orders of magnitude higher than in forage and concentrated feeds. Soil ingestion in farm animals occurs either as an involuntary ingestion due to soil adhering to vegetation or as an active ingestion in response to the lack of mineral elements. In ruminants, the ingestion of soil can supply more than 60% of trace minerals; it can represent up to 18% of the organic matter in cattle and even more than 50% in sheep [62].

Oral dosing or bathing with dilute cobalt solutions are satisfactory if the doses are regular and frequent. It is recommended to dose sheep twice a week with 2 mg of cobalt or once a week with 7 mg of cobalt and cattle with 5 to 10 times those amounts, depending on their size and age, to be totally adequate for severely deficient regions [12].

Although dietary supplements would be recommended, vitamin B12 injections are often given to cobalt-deficient animals, which usually respond rapidly to treatment, regaining appetite, vigor and weight [12]. It has even been used as an easy practical test to determine if there is a cobalt deficiency [12]. For the rapid correction of cobalt deficiency in cattle, the intramuscular administration of vitamin B12 at 500 to 3000 µg per head is recommended and can be repeated weekly [12], while, in lambs, the intramuscular administration of 100 µg per week or 150 µg every two weeks produces a rapid remission of all signs of deficiency and is as effective as cobalt administered orally at a rate of 7 mg per week [12]. Although parenteral injections of vitamin B12 prevent cobalt deficiency in ruminants, it is more convenient, and even economical, to supplement the diet with cobalt, allowing ruminal microorganisms to synthesize the vitamin for later absorption by the host [12].

Cobalt deficiency in ruminants can be prevented or cured by treating soils or pastures with fertilizers containing cobalt. In deficient areas, where grasses require regular fertilizer applications, adequate cobalt intake can be ensured by the inclusion of cobalt salts. However, the use of cobalt fertilizers is often impractical and uneconomical [12].

Cobalt can be supplemented with inorganic or organic sources. Several studies have evaluated multiple mixtures of organic minerals. Both cobalt chloride, nitrate or oxide, on the one hand, and cobalt carbonate and sulfate or Co glucoheptonate appear to be suitable sources of cobalt for ruminants [19,36,80]. However, the most important cobalt supplements are cobalt carbonate, a fine powder that contains more than 46% cobalt, cobalt sulfate, a fine granular crystal containing 21% cobalt, and cobalt sulfate monohydrate, a powder that contains 33% cobalt [12].

The addition of almost any source of Co at 40 mg/kg considerably increased the concentration of vitamin B12, vitamin B12 analogs and, consequently, the total corrinoids. Thus, the production of vitamin B12 was similar with the addition of 40 mg/kg of Co as sulfate, carbonate or glucoheptonate, while the addition of oxide of Co at 40 mg/kg of Co did not increase the production of vitamin B, compared to the cultures of the control [116]. On the other hand, the supplementation with 1 mg/kg of Co as the Co sulfate increased the concentration of vitamin B compared to other sources of Co, and also, the addition of the oxide to 1 mg/kg of Co almost doubled the production of the vitamin, which suggests that, although this is not the most available form of Co, it could be effective in ruminal granules used as long-term supplements [116].

Another strategy of providing cobalt, developed in Australia, is the use of a heavy pellet (bullet) administered orally [12,19], as well as cobaltous oxide pellets and controlled release glass pellets that contain cobalt, and that remain in the rumen and reticulum, to supply cobalt for long periods of time to grazing ruminants [19]. These ruminal boluses frequently provide multiple slow-release trace elements, including cobalt [12]. The granules can be lost by regurgitation [12,19] or become ineffective due to the formation of a surface layer of calcium phosphate [12]. The addition of a steel grinder, which provides an abrasive action, reduces the surface coating and extends the usefulness of the pellet [12].

*Saccharomyces cerevisiae* fermentation product supplements have been used, trying to verify if it could change the apparent ruminal synthesis and the duodenal flow of individual B vitamins in cows, verifying that it had no effect on the pattern of ruminal fermentation [117,118] and had no effect on vitamin B in dairy cows [65].

There is not much knowledge about the nutritional factors that drive the ruminal production of vitamin B12. For this reason, the idea of providing a preformed part of the vitamin B12 molecule, specifically 5,6-dimethylbenzimidazole (5,6-DMB), has been proposed to promote the apparent synthesis of the vitamin by ruminal bacteria [17].

Providing this precursor of CBL increased the apparent ruminal synthesis of CBL by ruminal bacteria by 34% (from 14.6 to 19.6 mg/day) but had no effect on various parameters related to ruminal fermentation; production or the plasma concentration of CBL (milk production and composition, protozoan count, ruminal pH, concentrations of volatile fatty acids and ammonia nitrogen in the ruminal content, intake, omasal flow and the ruminal digestibility of dry matter, matter organic, NDF, ADF and nitrogenous fractions) [17].

## 10. Conclusions

In conclusion, we have reviewed the role of cobalt in animal metabolism and, especially, in ruminants. It is important to note that cobalt does not have a known nutritional function, except as a component of vitamin B12, participating as coenzymes in two important enzyme systems: methylmalonyl CoA mutase, which requires adenosylcobalamin, and methionine synthetase, which requires methylcobalamin, and which are essential to obtaining energy through the ruminal metabolism. In ruminants, the efficacy of the microbial use of cobalt for the synthesis of cobalamin is influenced by multiple factors, such as the adequate contribution of cobalt, the ingredients and the composition of the diet and the composition of the ruminal microbiome and its effects on fermentation. To determine the cobalt status of the animal, the blood and liver levels of B12 can be assessed, but other very interesting options are the measurements of metabolites such as methylmalonic acid, homocysteine or holotranscobalamin in blood, as well as methylmalonic acid in urine. In general, the cobalt need is around 0.11 ppm (mg/kg), although it is recommended to increase this supplementation to 0.20 ppm (mg/kg), since the ruminal synthesis of vitamin B12 increases when the concentration of Co in the diet increases, and it has been proven that an increase in supplementation may be beneficial for ruminal fermentation, producing the maximum responses in milk production. For feedlot, although no significant effect on the digestibility of the diet or on the performance of the animals was shown, it was able to reduce their morbidity. In sheep, the requirements are much higher than in other species, so, for the growth of lambs, about 200 µg/day are required, and the deficiency causes a deterioration of the immune function and, therefore, a lower viability in newborn lambs.

## Figures and Tables

**Table 1:** Some experiences about supplementations with Co/vitamin B12 in dairy cattle and their most representative effects. BW: body weight, DM: dry matter and CBL: cyanocobalamin.

Action	Levels	Animals	Effect	References
Current cobalt requirements in ruminants	0.1 to 0.2 mg/kg	Dairy cattle	Non-available	[24]
Vitamin B12 supplementation	0.34 and 0.68 µg/kg BW	Dairy cattle	Non-available	[84]
Cobalt supplementation (Co-glucoheptanate)	2.1 mg/kg (dry period) and 1.1 mg/kg (lactation)	Dairy cattle	Increase milk production, weight loss and BCS; no effect on reproductive rates	[85]
Ration supplemented with Co (Co glucoheptonate)	3.6 ppm (mg/kg)	Dairy cattle	Increase hepatic concentrations of B12	[38]
Supplementation with Co	Increased 0 to 1 ppm (mg/kg)	Multiparous vs. primiparous cows	Increase milk production; no benefit for health of cows; no effect in first lactation	[5]
Ration supplemented with Co (Co-sulphate)	Extra supply Co (0.29-mg vs. 0.17-mg/kg DM)	Lactating dairy cows	No influence on ruminal parameters, nor characteristics of milk produced	[20]
Extra supplementation with cobalt	Oral, 0.13-mg vs. 0.27-mg Co/kg DM	Pregnant dairy cows	Slightly higher serum CBL concentrations	[18]
Supplementation dietary with Co	0.15, 0.89 or 1.71 mg/kg of Co, DM	Cows, antepartum period	Increase ruminal synthesis of vitamin B12 in colostrum and milk; no effect on intake of DM, production milk and milk components; no affect on hepatic or serum Co; increase Co in milk	[74]
Supplementation folic acid + vitamin B12	Injections, weekly	Dairy herds, different production levels	Better energy state: increased BCS and decreased fat and protein in early lactation; joint supplementation increases the effects	[30,42]
Supplementation folic acid + vitamin B12	Intramuscularly, weekly, 3 to 16 wk after calving	Multiparous cows in early lactation	Milk production increased 12%	[59]
Supplementation vitamin B12 (even adequate dietary Co-supply)	Intramuscular, weekly, 10 mg vitamin B12	Primiparous cows	Increases blood hemoglobin, B12 in milk and milk yield; decreases MMA serum	[30]
Supplements folic acid and vitamin B12 (combined)	Orally or parenterally	Multiparous cows, early lactation	increases production milk and milk components by improving the energy metabolism	[11,30,59]
Supplementation folic acid and vitamin B12 (alone or joint)	Intramuscular injections, weekly from 3 weeks	until 9 weeks after parturition	Increase folate and vitamin B12 in milk and liver; DM intake not affected; milk and lactose tended lower	[58]
Slow-release boluses (I, Se and Co)	Application intraruminal	Herd with a marginal iodine status	Milk production significantly higher (224 kg more milk)	[86]

**Table 2:** Some experiences about a supplementation with Co/vitamin B12 in feedlot and its most representative effects.

Action	Levels	Animals	Effect	References
Current cobalt requirements	0.10 y 0.11 mg/kg	Beef cattle	Non-available	[24,84]
Current cobalt requirements	0.15 y 0.25 mg/kg	Beef cattle	Non-available	[28,74,76,80]
Ration supplemented vitamin B	× 10, for pigs	Cross calves, stressed during transport	Not influence the weight gain or feed conversion; reduce morbidity	[24]
Co-deficient diet vs. Co-proper diet		Growing cattle	Co in plasma (108 vs. 271 pg/mL)	[80]
Diet supplemented with cobalt (Co sulphate)	0.07 to 0.69 mg Co/kg; estimated Co needs: 0.15 and 0.25-mg/kg ration	Bulls	Lower concentrations of vitamin B12 in plasma and liver; dietary Co inversely correlated homocysteine and MMA in plasma	[80,88]
Diet control vs. Co-supplemented	0.07 mg/kg vs. Co supplementation (0.12, 0.16 and 0.18 mg/kg)	Bulls, slaughtered	Lower weight, feed consumption and carcass weights (0.07 mg/kg); maximum growth (0.12 mg/kg); maximum feed intake (0.16 to 0.18 mg/kg)	[87]
Diet supplemented with cobalt (Co-chloride)	1 ys. 6 ppm (mg/kg)	Growing calves	No significant effect (digestibility, production, etc.)	[89]
Diets supplemented	0.22 ppm (mg/kg) of Co	beef cattle, growing	High liver Co values	[5]

## Abbreviations

ADF	acid detergent fiber
CBL	cyanocobalamin
Co	cobalt
DM	dry matter
EU	European Union
GIF	cobalamin-gastric intrinsic factor
HC	haptocorrin
HCT	homocysteine
holoHC	holohaptocorrin
holoTC	holotranscobalamin or active B-12
IF	intrinsic factor
MMA	methylmalonic acid
NDF	neutral detergent fiber
NRC	National Research Council
TC	transcobalamin
UMMA	urinary methylmalonic acid
VFA	volatile fatty acids
5,6-DMB	5,6-dimethylbenzimidazole
